# A Small Molecule Dual Inhibitor of *agr* Quorum Sensing and ATP Synthase Blocks Acute and Chronic Virulence Phenotypes in *Staphylococcus aureus*

**DOI:** 10.1021/acsinfecdis.6c00432

**Published:** 2026-09-23

**Authors:** Troy D. Vulpis, Jordan T. York, A.P. Clay, Thomas J. Polaske, Samuel W. Markovich, Kijeong Lee, Douglas H. Chang, Sean P. Palecek, David M. Lynn, John-Demian Sauer, Lloyd M. Smith, Helen E. Blackwell

**Affiliations:** † Department of Chemistry, 5228 University of Wisconsin−Madison, 1101 University Ave., Madison, Wisconsin 53706, United States; ‡ Department of Medical Microbiology and Immunology, University of Wisconsin−Madison, 1550 Linden Dr., Madison, Wisconsin 53706, United States; § Department of Chemical and Biological Engineering, University of Wisconsin−Madison, 1415 Engineering Dr., Madison, Wisconsin 53706, United States

**Keywords:** accessory gene regulator system, antibiotic resistance, antivirulence, cell−cell signaling, persisters, small molecule probe

## Abstract

The accessory gene regulator (*agr*) quorum-sensing system in *Staphylococcus aureus* plays a prominent role in acute infections caused by this common pathogen. In contrast, ATP synthase affects the ability of *S. aureus* to establish chronic infections and persist as small colony variants. In this study, we report that a synthetic small molecule, CP-20, is capable of dually inhibiting *agr* and ATP synthase. We demonstrate CP-20’s ability to counteract key virulence phenotypes associated with both pathways, including hemolysis, biofilm formation, and antibiotic resistance. We also show that CP-20 facilitates more efficient clearance of *S. aureus* by murine macrophages than genetic inactivation of either *agr* or ATP synthase alone, suggesting that dual inhibition may have an additive effect on ablating intracellular survival. This work demonstrates the utility of a single chemical modulator to attenuate *S. aureus* pathogenesis via two nonessential pathways and suggests a pathway toward nonbactericidal virulence control.

## Graphical abstract

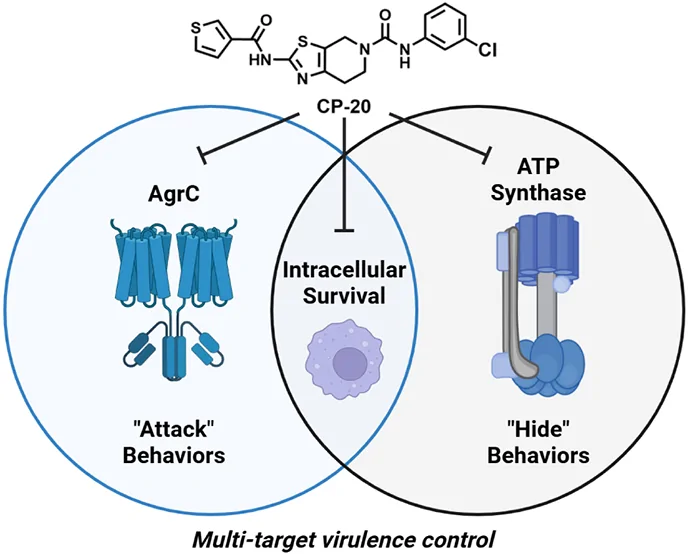

## Introduction

*Staphylococcus aureus* is a Gram-positive bacterium that is a major economic, industrial, and healthcare burden worldwide.^1–3^ This common pathogen is best known for its role in dangerous antibiotic-resistant infections, such as those caused by methicillin-resistant *S. aureus* (MRSA) that are responsible for over 100,000 deaths per year globally.^4^ While it is often associated with skin and soft tissue infections, *S. aureus* can cause both acute and chronic illness by targeting many other human tissues and organs (e.g., gastroenteritis, bacteremia and associated sepsis, pneumonia, endocarditis, osteomyelitis, etc.).^5^ To survive and persist in hosts, *S. aureus* uses a strategy called phenotypic switching, where the bacterium switches between “attack” and “hide” lifestyles.^6–12^ “Attack” phenotypes are characterized by highly virulent toxin and exoprotein production used for tissue invasion, while the “hide” lifestyle is typified by metabolically deficient and slow-growing small colony variants (SCVs) that are resistant to multiple classes of antibiotics and less likely to evoke an immune response than their “attack” counterparts. In general, this phenotypic switching strategy is beneficial as it allows a genetically identical bacterial population to adapt to complex and changing environments found in host systems, thereby conferring a survival advantage. Notably, phenotypic switching by *S. aureus* has been suggested to play a critical role in chronic and recalcitrant infections in which long periods of benign symptoms are interrupted by problematic relapses.^7,10^

The increasing prevalence of antibiotic-resistant *S. aureus* strains has sparked the development of so-called “anti-virulence” approaches aimed at inhibiting either “attack” or “hide” behaviors.^13–16^ These strategies typically involve targeting systems that are not essential for cell growth, thereby curbing problematic phenotypes without providing selective pressure for new resistances to develop. Antivirulence strategies targeting *S. aureus* “attack” phenotypes have frequently focused on the accessory gene regulator (*agr*) quorum sensing (QS) system due to its regulatory influence on a broad set of virulence factors.^17,18^ A schematic of this chemical signaling system is shown in Figure 1A. The *agr* system enables *S. aureus* to coordinate group attacks by sensing signals called autoinducing peptides (AIPs) that accumulate at high cell density. The AIPs are produced at a low basal level via the processing of the AgrD propeptide via the transmembrane peptidase AgrB (and in certain strains MroQ) and exit the cell via an unknown mechanism.^19–21^ Once a quorate *S. aureus* population has amassed, the AIP signal will achieve sufficient local extracellular concentration to bind to and activate its cognate receptor, the transmembrane histidine kinase AgrC, causing the autophosphorylation of its intracellular kinase domain.^22^ AgrC then transfers this phosphate to its partner cytoplasmic response regulator, AgrA, enabling AgrA to bind to several *agr*-associated promoters and upregulate the expression of an arsenal of secreted toxins, exoenzymes, and superantigens that are required for invasion and pathogenesis, along with the *agr* machinery.^17,18,23–26^ Supporting *agr* as an antivirulence approach, animal models of dermonecrosis, atopic dermatitis, and osteomyelitis have demonstrated that *agr* inactivation can significantly reduce key indications of acute *S. aureus* infection.^27–30^

**Figure 1:**
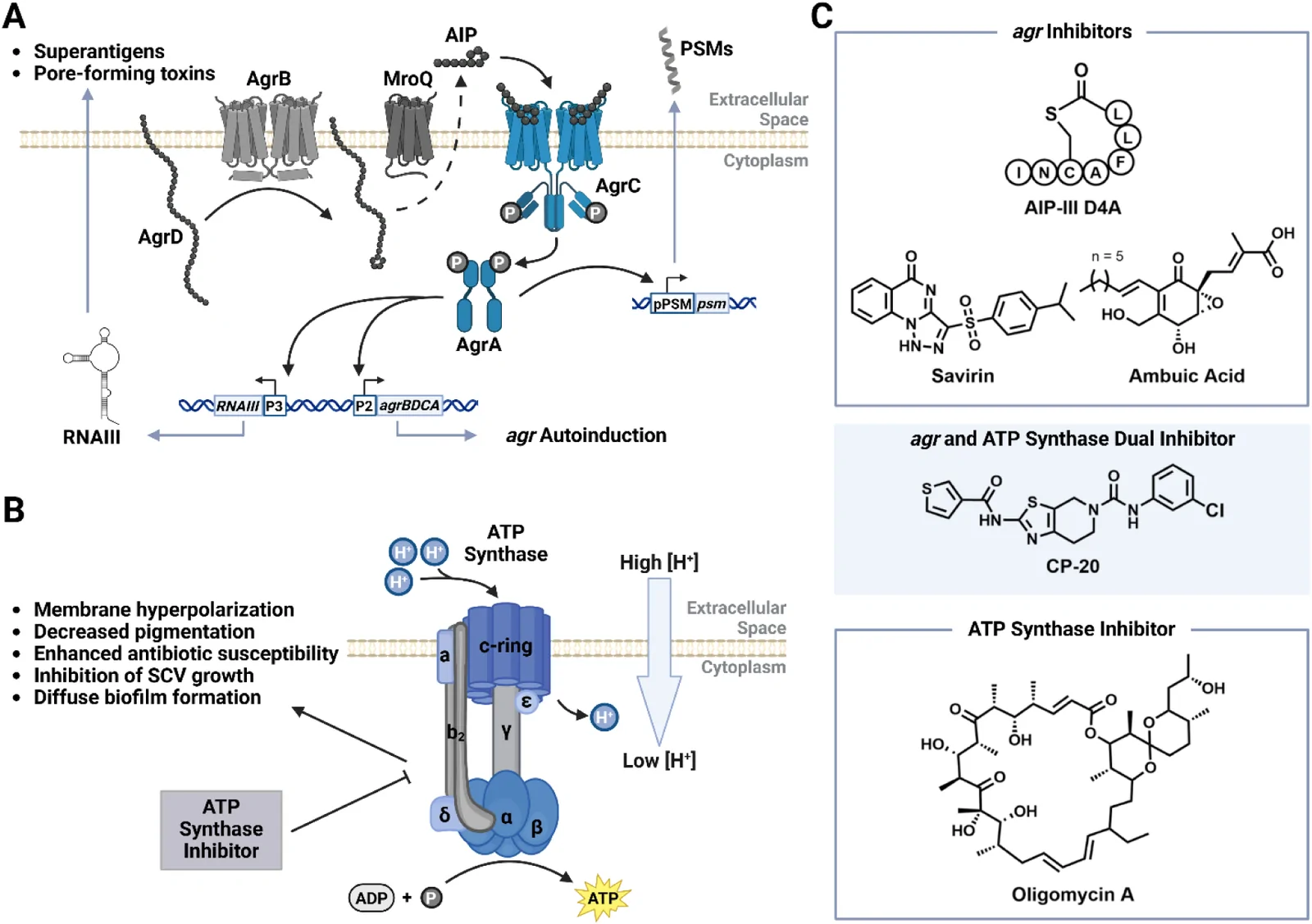
Regulation of *agr* and ATP synthase in *S. aureus*. (A) Schematic of the *agr* quorum-sensing system in *S. aureus*. (B) Schematic of ATP synthase and its function with regard to downstream virulence phenotypes. (C) Structures of selected previously reported inhibitors of the *agr* system and ATP synthase, along with CP-20.

Interest in agents capable of blocking *agr* has grown since the discovery of AIP signals.^31^ Accordingly, the majority of the chemical *agr* inhibitors discovered to date are close AIP analogs and peptidomimetics that interfere with the AIP-AgrC interaction. Early generations of these AIP mimics, like AIP-III D4A (Figure 1C), were produced by altering a naturally occurring AIP scaffold by a single residue^32,33^ or via truncation of the exocyclic tail.^34,35^ Relatively few small molecules have been identified to date that interfere with *agr*; prominent examples include savirin that acts on AgrA and ambuic acid that blocks AIP biosynthesis.^36,37^ Recently, we reported the discovery of a synthetic small molecule *agr* inhibitor, CP-20 (Figure 1C), via high-throughput screening^38^ and determined that it can block *agr* via competitive binding to AgrC.^39^ Several of these peptide and small molecule *agr* modulators have been shown to be efficacious at blocking acute infection in mouse models.^36,37,40^ However, in chronic infection contexts in which the “hide” lifestyle is more prevalent, *agr* inhibition, either chemically or genetically, may provide *S. aureus* with a competitive growth advantage. For example, strains lacking a functional *agr* system have been shown to survive within human lung epithelial cells at higher rates than their wild-type counterparts, suggesting that *agr* dysfunction may contribute to the development of chronic infections.^41^ Others have found that *S. aureus* *agr* mutants are more frequently observed in hospital settings, further supporting the notion that *agr* dysfunction may provide a fitness advantage in certain environmental and infection contexts.^42^ Thus, the development of complementary antivirulence approaches to existing *agr* inhibition strategies that avoid this shift toward the “hide” lifestyle in *S. aureus* is of interest. Such approaches would readily extend to related species, such as *Staphylococcus epidermidis*, that leverage “hide” phenotypes (e.g., biofilm formation) to persist in chronic infections.^43^

Many of the existing chemical probes that target “hide” lifestyles in *S. aureus* inhibit specific behaviors associated with persistent infections, such as antioxidant pigment production or antibiotic resistance, rather than a global regulator like *agr*. Such compounds include the small molecules BPH-652 and naftifine, which inhibit staphyloxanthin biosynthesis via CrtM and CrtN, respectively, thereby rendering *S. aureus* more susceptible to lethal oxidative stress.^44,45^ Other examples include MAC-545496 and verteporfin, which increase *S. aureus*’ susceptibility to killing by β-lactam antibiotics and cationic antimicrobial peptides, respectively, by inhibiting the GraSR system.^46,47^ Given the wide variety of strategies that *S. aureus* uses to persist in the host, a global approach may be more effective at interfering with the “hide” lifestyle.^9,48^ One such global target is the alternative sigma factor, SigB, which regulates stress response and SCV formation, thereby enabling chronic infection by *S. aureus*.^8^ To our knowledge, the plant natural product rhodomyrtone is the only reported inhibitor of SigB, although its mechanism of SigB inhibition is unknown.^49^ However, rhodomyrtone is a known antibiotic against *S. aureus*, and concentrations needed to observe effects on SigB are in the same regime as those needed to kill cells. Thus, the continued search for nonbactericidal probes that interfere with the “hide” lifestyle is still warranted.

ATP synthase has gained considerable attention as an antibiotic target in obligate aerobes, such as *Mycobacterium tuberculosis*.^50^ In contrast, *S. aureus* does not require ATP synthase for growth, as it can shift toward fermentation as an alternate means of generating ATP.^51^ Several studies have linked a functional ATP synthase to “hide” phenotypes in *S. aureus* like aminoglycoside and antimicrobial peptide resistance, antioxidant pigment production, biofilm formation, and SCV viability (Figure 1B).^52–55^ Thus, ATP synthase inhibition may provide a nonbactericidal means of interfering with multiple aspects of chronic *S. aureus* infection. ATP synthase is made up of eight distinct protein subunits, assembled with the typical stoichiometry α_3_β_3_γδεab_2_c_10_, all of which are essential for proper function.^56^ Numerous ATP synthase inhibitors have been identified in both prokaryotes and eukaryotes, many of which are active in *S. aureus* (e.g., oligomycin A, tomatidine, and venturicidin A) due to the high degree of conservation of ATP synthase across all kingdoms of life (Figure 1C).^51,52,57,58^ Certain of these compounds are known to target specific components of ATP synthase complex (e.g., oligomycin A targets the c-ring).^59^ Studies using ATP synthase mutants and inhibitors have collectively linked ATP synthase activity to pathogenicity *in vitro* and *in vivo*, validating ATP synthase as an antivirulence target in *S. aureus*.^54,55,60,61^ Given *S. aureus* *’* ability to pivot between acute invasion and the SCV lifestyle, molecules that target pathways controlling both of these infection modes represent highly desirable probes to evaluate the prospect of manipulating these systems to gain favorable health outcomes.

Herein, we report that that the small molecule AgrC inhibitor, CP-20, also inhibits ATP synthase in *S. aureus*, establishing it as the first dual inhibitor of these two systems. We outline our discovery process and show that CP-20 blocks both *agr*- and ATP synthase-associated virulence phenotypes (e.g., hemolysis, antibiotic resistance, biofilm formation, etc.) and renders *S. aureus* highly susceptible to clearance in a macrophage infection assay. This study illustrates CP-20’s value as a chemical modulator of both acute and chronic pathogenicity in *S. aureus*. We anticipate that this probe and the insights provided herein will enable further investigation into *S. aureus*’ phenotypic flexibility by providing a means of preventing *S. aureus* from toggling between “attack” and “hide” lifestyles. Furthermore, by blocking both *agr* and ATP synthase, CP-20 provides a chemical link between virulence regulation and central metabolism and thus may help to elucidate the synergistic or antagonistic interactions between these two distinct cellular functions. These insights will improve the understanding of the contexts in which *S. aureus* relies on its functional plasticity to respond to environmental changes and persist in the host and provide a multifaceted pathway toward virulence control.

## Results

### CP-20 Induces a Dramatic Loss of Golden Pigment in *S. aureus*

During our prior studies of CP-20,^39^ we observed that it not only inhibited *agr* but also caused a dramatic loss of golden pigment in *S. aureus* upon treatment (Figure 2A). This pigment is a distinguishing characteristic of *S. aureus*, and its loss (in the absence of any appreciable growth inhibition up to 100 μM; Figure S1) suggested to us that CP-20 could modulate targets beyond *agr*. We suspected that the stark color change resulted from decreased production of the carotenoid pigment, virulence factor, and antioxidant, staphyloxanthin (Figure 2B). Staphyloxanthin is an important contributor to the *S. aureus* infection lifestyle, as it functions to neutralize reactive oxygen species generated by the host immune system, thereby protecting *S. aureus*, and inhibition of its biosynthesis has been suggested as a potential antivirulence strategy (see above).^44,45,62,63^

**Figure 2:**
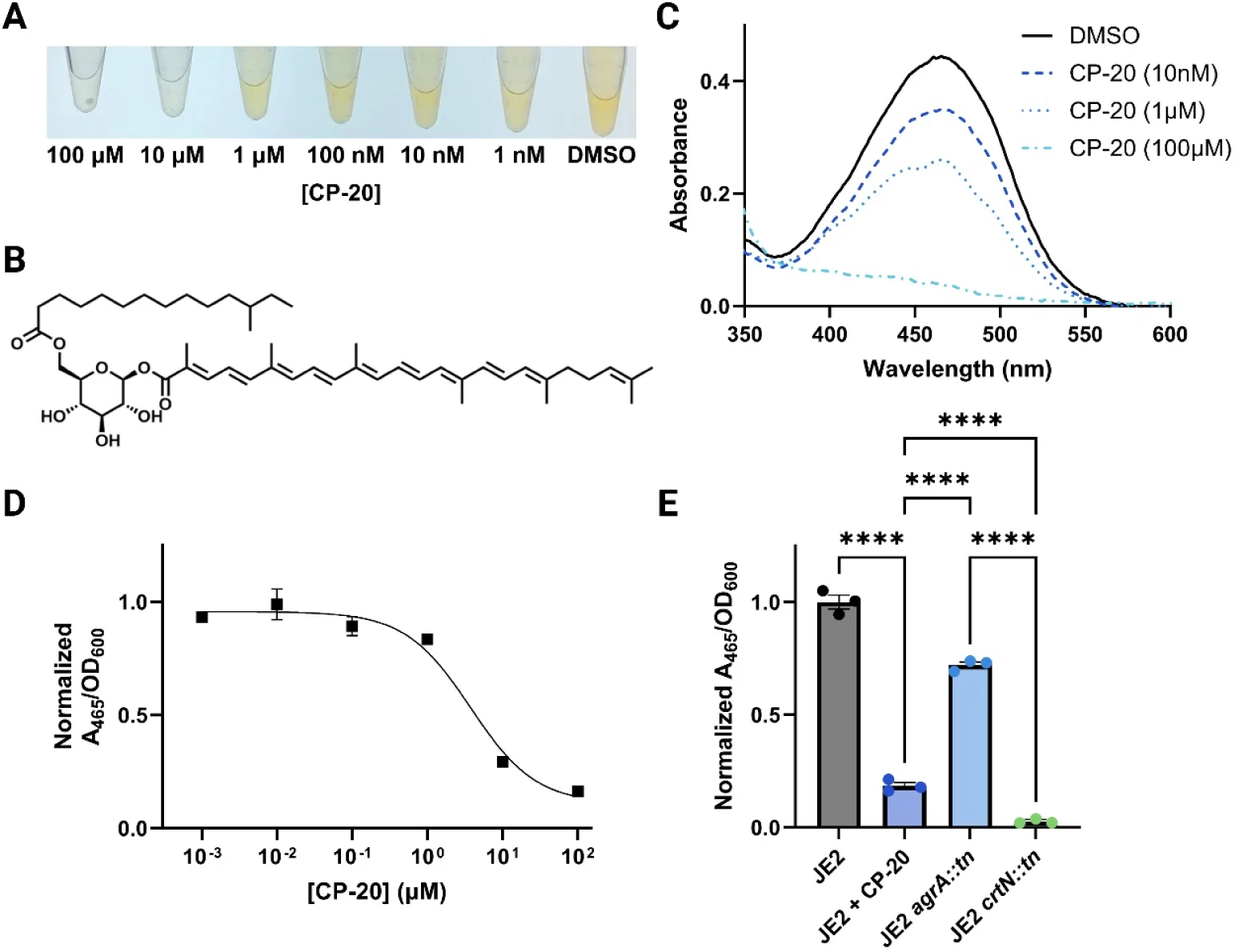
CP-20 induces a dramatic loss of golden pigment in *S. aureus*. (A) Representative images of methanol extracts of *S. aureus* JE2 cell pellets. Cells were treated with CP-20 at varying concentrations prior to pelleting and methanol extraction. (B) Chemical structure of staphyloxanthin. (C) Absorbance spectra of methanol extracts of *S. aureus* JE2 cell pellets. Cells were treated with CP-20 at varying concentrations prior to pelleting and methanol extraction. (D) Relative staphyloxanthin quantification in *S. aureus* JE2 following treatment with CP-20 in a dose–response format. Staphyloxanthin quantified by normalizing absorbance at 465 nm (A_465_) to cell growth (OD_600_). (E) Relative staphyloxanthin quantification for wild-type *S. aureus* JE2, JE2 treated with 25 μM CP-20, JE2 *agrA::tn*, and JE2 *crtN::tn*. Staphyloxanthin was quantified by normalizing A_465_ to OD_600_. Data in panels D and E are shown as mean ± SEM for three biological replicates. **** α < 0.0001 by an ordinary one-way ANOVA with Tukey’s multiple comparisons test.

To determine if CP-20 caused a reduction in staphyloxanthin levels, we treated *S. aureus* strain USA300 JE2 with varying concentrations of CP-20 for 24 h, extracted the cell pellets into methanol, and collected absorbance spectra (300–800 nm) of these samples. In untreated samples, we observed an absorbance peak at approximately 465 nm that is characteristic of staphyloxanthin-containing extracts (Figure 2C).^64^ Treatment with CP-20 led to a dose-dependent decrease in absorbance at 465 nm (Figure 2D). To test if this loss of pigment resulted from CP-20’s *agr* inhibition, we quantified pigment production in a *S. aureus* JE2 strain containing a transposon mutation in the *agrA* gene (JE2 *agrA::tn*) and compared it to a strain deficient in staphyloxanthin biosynthesis (JE2 *crtN::tn*).^65^ JE2 *agrA::tn* demonstrated a modest reduction in pigment relative to JE2, but approximately 3-fold less than that observed when JE2 was treated with 25 μM CP-20 (28% vs 82%; Figure 2D-E). Neither CP-20 treatment nor genetic *agr* inactivation completely blocked staphyloxanthin production, as was observed in JE2 *crtN::tn*. These results suggested that CP-20 may target other systems beyond *agr*, and that these additional targets are likely to impact pigment production.

### CP-20 Renders *S. aureus* More Susceptible to Antibiotic Treatment

Given the role of staphyloxanthin in *S. aureus*’ response to oxidative stress, we hypothesized that CP-20 may affect *S. aureus*’ ability to respond to other stressors, such as antibiotics. To address this question, we conducted broth microdilution experiments with a representative panel of antibiotics and evaluated their minimum inhibitory concentrations (MICs) in the presence and absence of CP-20. Co-treatment with CP-20 led to 4- and 8-fold decreases in MIC for gentamicin and polymyxin B in *S. aureus* JE2, respectively, relative to untreated control (Table 1). Again, CP-20 alone did not inhibit cell growth at the concentration assessed in these assays (25 μM; Figure S1), suggesting that CP-20 functions as an adjuvant for these two antibiotics. Untreated JE2 *agrA::tn* exhibited the same susceptibility as wild type JE2 to all antibiotics tested, indicating that the increased susceptibility observed upon CP-20 treatment was independent of *agr* activity (Table 1). CP-20’s adjuvant activity also extended to the closely related pathogen *S. epidermidis*, wherein cotreatment of CP-20 decreased the MIC of polymyxin B 4-fold (Table S1). In a prior study of the *S. aureus* JE2 transposon mutant library, Vestergaard et al. found that mutations to the GraSR and VraFG two-component systems and to ATP synthase caused similar shifts in MIC for these two classes of antibiotics.^52^ In view of this past result, we reasoned that GraSR/VraFG and ATP synthase could represent possible targets for CP-20 involved in its non-*agr* activity.

**Table 1:** Minimum Inhibitory Concentrations (MIC_90_’s) for Selected Antibiotics against *S. aureus* JE2 and Associated Transposon Mutants in the Presence and Absence of CP-20 (at 25 μM)

Strain ▶	JE2	JE2 + CP-20	JE2 *atpA::tn*	JE2 *agrA::tn*
Antibiotic ▼	MIC_90_ (μg/mL)	MIC_90_ (μg/mL)	MIC_90_ (μg/mL)	MIC_90_ (μg/mL)
Polymyxin B	128	16	16	128
Gentamicin	1	0.25	0.0625	1
Vancomycin	2	2	2	2
Oxacillin	2	2	2	2
Linezolid	2	2	2	2

### CP-20 Inhibits ATP Synthase

We conducted a label-free bottom-up proteomics experiment to begin to evaluate the effects of CP-20 treatment on the *S. aureus* JE2 proteome and narrow our focus to its biomolecular targets (Figure S2 and Supplemental Data 1). In this experiment, JE2 was grown to exponential phase in the presence of CP-20 (10 μM) or vehicle control, and relative differences in protein abundance between these two samples were determined with label-free quantification.^66^ As expected, given CP-20’s activity toward *agr* quorum sensing, we identified several differentially abundant proteins (*p* < 0.05) in CP-20-treated samples that are characteristic of an inactive *agr* system (e.g., AgrB/AgrC/AgrA, Rot, AtlA, and Hla).^23^ We also observed the abundance of several proteins to change in response to CP-20 treatment that suggested ATP synthase dysfunction (e.g., proteostasis machinery ClpB, GroEL/GroES, RocF, and Adh); this set of proteins is aligned with that reported in a recent proteomics study on a *S. aureus* ATP synthase mutant.^23,54^ Conversely, we found that GraS and GraR protein levels were unchanged across untreated or CP-20-treated samples. We conducted analogous proteomics experiments with the JE2 *agrA::tn* and JE2 *atpA::tn*, finding that a large portion of the 276 proteins with differential abundance in CP-20-treated cells exhibited similarly altered abundance in the *agr* (78 proteins) and ATP synthase (109 proteins) single mutant strains (Figure S2). We note that these preliminary experiments had relatively limited coverage of the membrane-associated and secreted proteomes and thus may have omitted key proteins for which expression is impacted by CP-20. Nevertheless, these results provided initial support that CP-20 may block ATP synthase as a secondary target.

We next evaluated CP-20’s activity in a cell-based transcriptional reporter developed by Rapun-Araiz et al. in which GFP production is under the control of the GraSR-regulated *mprF* promoter.^67^ CP-20 had no activity in this system, further suggesting that it likely does not target GraSR; these data are shown in Figure 3A alongside analogous GFP reporter data for AgrCA for comparison.^67^ The GraSR and VraFG systems are highly interconnected, with VraFG functioning to activate GraR-regulated transcription by signaling through the histidine kinase GraS.^68^ Therefore, we reasoned that CP-20 is likely also inactive toward VraFG, as VraFG inhibition should also decrease *mprF* transcription via GraR in this reporter assay (both GraSR and VraFG are present in this reporter strain).

**Figure 3:**
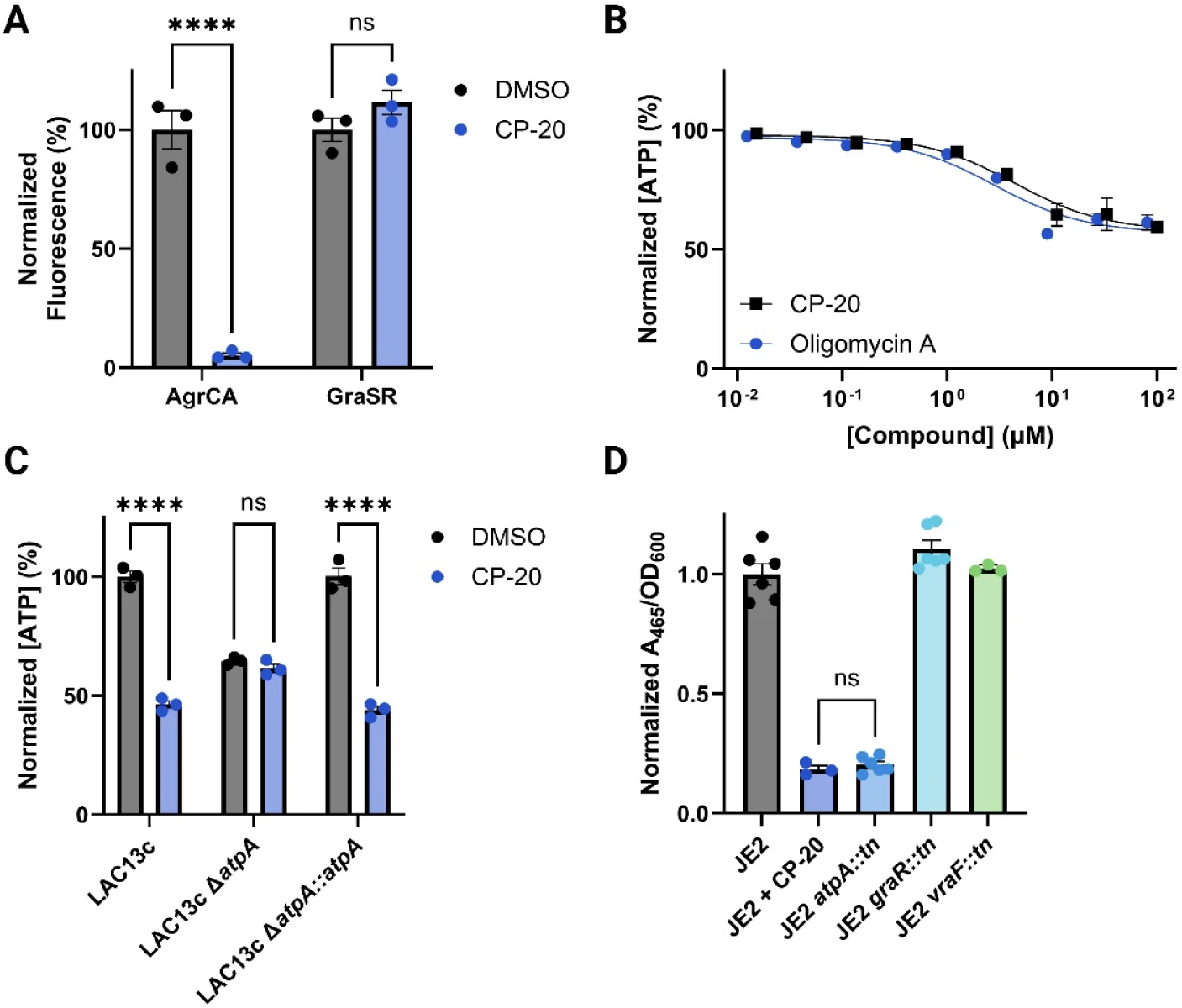
CP-20 inhibits ATP synthase. (A) Fluorescent transcriptional reporter data for the AgrCA and GraSR two component systems treated with DMSO (vehicle control) or CP-20 (10 μM). (B) ATP quantification of *S. aureus* treated with CP-20 and the known ATP synthase inhibitor, oligomycin A. Relative ATP concentrations were determined using BacTiter-Glo following manufacturer’s instructions. (C) ATP quantification in wild-type *S. aureus* (LAC13c), Δ*atpA*, and Δ*atpA::atpA* genetic backgrounds. Each strain was treated with DMSO (vehicle control) or CP-20 (10 μM). Relative ATP concentrations were determined using BacTiter-Glo following manufacturer’s instructions. (D) Relative staphyloxanthin quantification for untreated *S. aureus* JE2, CP-20 (25 μM)-treated JE2, JE2 *atpA::tn*, JE2 *graR::tn*, and JE2 *vraF::tn*. CP-20-treated condition reproduced from Figure 2E. Staphyloxanthin was quantified by normalizing absorbance at 465 nm (A_465_) to cell growth (OD_600_). For all graphs, data are shown as mean ± SEM for at least three biological replicates. **** α < 0.0001 by a two-way ANOVA with Šídák’s multiple comparisons test (panels A and C). A Student’s *t* test was conducted in panel D.

Next, we evaluated CP-20’s activity toward ATP synthase by adapting an assay developed by Yarlagadda et al. that uses BacTiter-Glo to measure changes in intracellular ATP levels.^57^ We found that CP-20 decreased cellular ATP in a dose-dependent manner (IC_50_ = 4.40 μM), similar to the known ATP synthase inhibitor oligomycin A (IC_50_ = 2.83 μM) (Figure 3B). Treatment of a *S. aureus* Δ*atpA* mutant (*atpA* encodes the ATP synthase α subunit) with CP-20 had no additional effect on relative ATP levels; CP-20’s activity was restored upon genetic complementation with the *atpA* gene (Δ*atpA::atpA*).^54^ This result indicates that CP-20 relies on a functional ATP synthase to induce changes in intracellular ATP (Figure 3C). We also found that JE2 *atpA::tn* produced a low level of staphyloxanthin similar to that of CP-20-treated cells, further supporting this target assignment (Figure 3D). In contrast, the *graR* and *vraF* transposon mutants exhibited no change in pigmentation. Collectively, these results are congruent with CP-20 inhibiting ATP synthase activity in *S. aureus*.

We were curious whether CP-20 may interfere with the cell membrane, despite not having appreciable antibiotic activity in *S. aureus*, and that membrane interference could contribute to its ability to inhibit the activity of the transmembrane ATP synthase complex. To test this hypothesis, we measured *S. aureus* membrane permeability in the presence of CP-20 using the non-cell-penetrant nucleic acid-targeting dye, SYTOX. Using DMSO and Triton X-100 (0.1% v/v) as negative and positive controls for membrane permeabilization, respectively, we determined that CP-20 can disrupt the *S. aureus* membrane at concentrations (≥6.3 μM) required to inhibit ATP synthase (Figure S3A). However, the JE2 *atpA::tn* mutant did not show the same degree of membrane disruption as CP-20, suggesting that CP-20’s activity toward the membrane is not likely derived from its inhibition of ATP synthase (Figure S3B). To further examine the connection between membrane disruption and ATP synthase inhibition, we evaluated a CP-20 analog, CP-20-I, which replaces the *meta*-substituted chlorine in CP-20 with an iodine (Figure S3C).^39^ CP-20-I is approximately 5-fold more potent than CP-20 in *agr* inhibition assays, and we were interested in whether it also exhibited different levels of membrane disruption and/or bioactivity toward ATP synthase. Interestingly, CP-20-I disrupted the membrane to a lesser extent (18% lower fluorescence signal in SYTOX assay) and was 10% less efficacious toward ATP synthase than CP-20. CP-20-I also showed less cytotoxicity in two mammalian cell lines (3T3 and HUVEC) when compared with CP-20 (Figure S4), which may derive from CP-20-I’s reduced membrane disruption and ATP synthase activities. Despite its reduced activity in these ATP synthase-related assays, CP-20-I retains similar antibiotic adjuvant activity to CP-20 (Table S1), suggesting that the CP-20 scaffold may be further tuned to improve its bioactivity while limiting mammalian cytotoxicity. Together, these data suggest that membrane disruption may contribute to CP-20’s ability to inhibit ATP synthase (e.g., via altering its positioning in the membrane, structure, stability, etc.). Such a mechanism has been demonstrated for the *Escherichia coli*-targeting antibiotic EcDBS1R4, which inhibits ATP synthase by sequestering cardiolipin molecules from other lipids in the ATP synthase membrane microenvironment.^69^

### CP-20 Abrogates Both Acute and Chronic Virulence Phenotypes

We reasoned that the dual inhibition of AgrC and ATP synthase by CP-20 could interfere with both acute and chronic virulence phenotypes in *S. aureus*, as introduced above. The *agr* system enables *S. aureus* to activate an arsenal of virulence factors, including the production of pore-forming toxins capable of lysing host red blood cells (RBCs), such as α-hemolysin.^18^ This phenotype is readily measurable *in vitro* by monitoring the lysis of rabbit RBCs by *S. aureus* cell-free supernatants. We observed untreated wild type *S. aureus* JE2 to readily lyse RBCs, but treatment with CP-20 completely ablated hemolytic activity, phenocopying JE2 *agrA::tn* (Figure 4A). JE2 *atpA::tn* exhibited a modest reduction in hemolysis relative to untreated JE2, but not to the same extent as CP-20-treated cells or JE2 *agrA::tn*. This result is consistent with previous reports showing that ATP synthase inactivation can lead to partial reductions in hemolysis (likely due to decreased ATP availability to support AgrC phosphorylation), but this phenotype was not completely ablated, as in the *agr* mutant.^61^ *S. aureus* also produces detergent-like peptide toxins called phenol-soluble modulins (PSMs) that are under positive *agr* control.^70^ δ-toxin is an α-type PSM that can both trigger host inflammatory responses via the FPR2 receptor and form transient pores in host membranes in an FPR2-independent manner, making it an acute virulence factor of concern.^70^ We quantified δ-toxin levels in cell supernatant using liquid chromatography–mass spectrometry and found that treatment with CP-20 completely blocked δ-toxin production in wild type JE2 (Figure 4B). JE2 *atpA::tn* demonstrated a minor decrease in δ-toxin production relative to JE2, similar to what was observed in the hemolysis assay. CP-20 treatment (at 10 μM) further reduced δ-toxin levels to baseline in JE2 *atpA::tn*, indicating that CP-20 affects this phenotype in an ATP synthase-independent manner and that its activity toward *agr* is required for complete inhibition of δ-toxin production.

**Figure 4:**
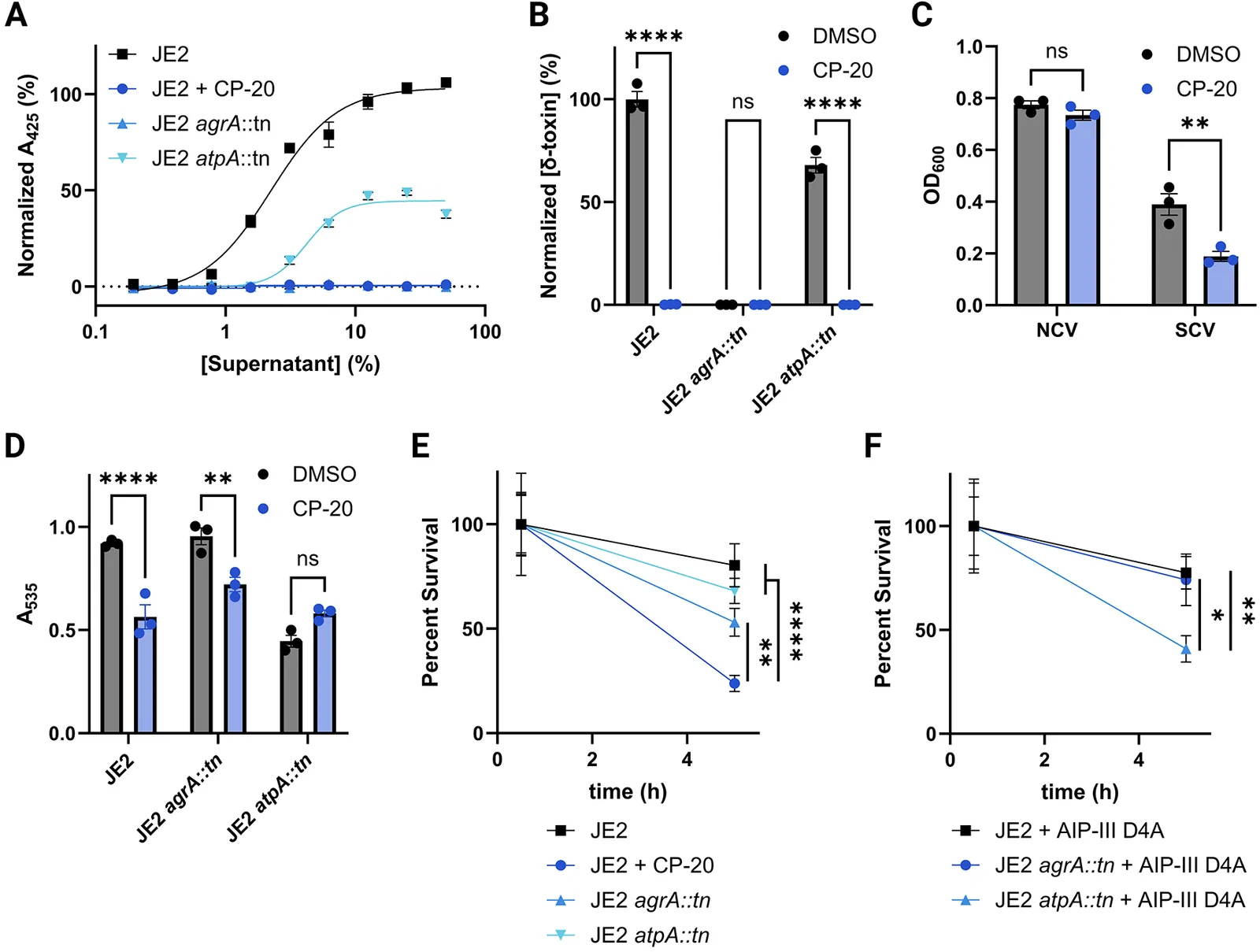
CP-20 abrogates both acute and chronic virulence phenotypes. (A) Rabbit red blood cell (RBC) hemolysis measurements. RBCs were treated with supernatant from wild type *S. aureus* (JE2) ± CP-20 (10 μM) and select transposon mutants. Absorbance at 425 nm (A_425_) is measured as a proxy for hemolysis. (B) δ-toxin quantification using LC-MS. All strains were treated with either DMSO (vehicle control) or CP-20 (10 μM). (C) Cell growth measurements (OD_600_) for normal colony variant (NCV) and HQNO-induced small colony variant (SCV) *S. aureus* in response to treatment with CP-20 (25 μM). (D) Safranin staining-based biofilm measurements of biofilms formed by JE2, JE2*agrA::tn*, and JE2*atpA::tn*. Absorbance at 535 nm is used as a proxy for total adherent biomass. All strains were treated with either DMSO (vehicle control) or CP-20 (10 μM). (E-F) Survival of *S. aureus* treated with CP-20 (25 μM) or AIP-III D4A (100 nM) in *in vitro* macrophage killing assays. Cells were treated with DMSO (vehicle control) unless otherwise noted. For all plots, data are shown as mean ± SEM for at least three biological replicates. * α < 0.05, ** α < 0.01, *** α < 0.001, **** α < 0.0001 by a two-way ANOVA with Šídák’s multiple comparisons test (panels B–D). *p < 0.05, **p < 0.01, ****p < 0.0001 by a Student’s *t* test (panels E-F).

As noted above, and in contrast to *agr*, ATP synthase activity is more frequently associated with chronic infection phenotypes in *S. aureus*, such as the persistence of respiration-deficient small colony variants (SCVs). A prior study of the ATP synthase inhibitor tomatidine revealed it to have selective bacteriostatic activity toward *S. aureus* SCVs, suggesting a potential strategy for combating these resilient subpopulations.^55^ We hypothesized that CP-20 may similarly inhibit the growth of SCVs. To test this hypothesis, we induced respiration-deficient SCV formation by treating *S. aureus* JE2 with 2-heptyl-4-hydroxyquinoline-*N*-oxide (HQNO) and measured CP-20’s effect on cell growth. CP-20 treatment led to a 51% reduction in OD_600_ of HQNO-treated SCVs, but did not affect the growth of the untreated, normal colony variant control (Figure 4C, Figure S5). This result suggests that CP-20 can selectively inhibit the growth of respiration-deficient SCVs and is consistent with prior studies of tomatidine’s selective antibiotic effects.^55^

### CP-20 Inhibits Biofilm Formation in an *agr*-Independent Manner

*S. aureus* can form robust biofilms that are prevalent in many chronic infections, including those associated with implanted medical devices.^71^ These biofilms are sensitive to both *agr* and ATP synthase activities. Chemical inhibition and genetic inactivation of the *agr* system can increase *S. aureus* biofilm levels when grown under flow, presumably due to the diminished production of PSMs and extracellular proteases and the heightened expression of surface attachment proteins.^71^ In other contexts, *agr* mutants have been shown to have varied effects on biofilm formation that depend on growth conditions (e.g., static versus dynamic) and surface (e.g., treated versus untreated microtiter plate).^72,73^ In contrast, *S. aureus* ATP synthase mutants tend to form more diffuse biofilms than their wild type counterparts, enabling more efficient clearance by immune cells.^54^ Given the seemingly opposing roles of *agr* and ATP synthase on biofilm formation, we were interested in examining the effects of CP-20—a chemical inhibitor of both systems—on *S. aureus* biofilm formation. To start, we used a safranin staining-based assay to quantify biofilm formed by wild type JE2 and the *agrA* and *atpA* transposon mutants in microtiter plates. JE2 and JE2 *agrA::tn* formed similar amounts of biofilm in this assay. In contrast, JE2 *atpA::tn* was significantly impaired in biofilm formation (∼55% reduction) relative to JE2 (Figure 4D). We then performed this assay in the presence of *S. aureus* AIP-I (native *agr* agonist) or AIP-III D4A (an *agr* inhibitor^32^). We expected that treatment with AIP-I would activate the *agr* system and thus inhibit biofilm formation in wild type *S. aureus*, but not in an *agr* mutant. Indeed, treatment with AIP-I led to a 34% reduction in biofilm in JE2 but had no effect on biofilm formation in JE2 *agrA::tn* (Figure S6). In contrast, the *agr* inhibitor AIP-III D4A had no significant effect on biofilm in either wild type or *agrA::tn* (Figure S6). We next examined the effects of CP-20 in this biofilm assay. Treatment with CP-20 led to a 40% reduction in biofilm in *S. aureus* JE2 relative to a DMSO control (Figure 4D). However, and unlike either AIP-I or AIP-III D4A, CP-20 reduced biofilm formation in the absence of a functional *agr* system (i.e., JE2 *agrA::tn*) (Figure 4D), suggesting that its inhibitory effects on biofilm are *agr*-independent. In turn, treatment with CP-20 had no effect on biofilm formation in the ATP synthase mutant (i.e., JE2 *atpA::tn*, Figure 4D). This result supports that CP20’s antibiofilm activity in this assay derives from its inhibition of ATP synthase.

### CP-20 Renders *S. aureus* Highly Susceptible to Clearance by Macrophages

*S. aureus* relies on both a functional *agr* system and ATP synthase to optimally evade host immune responses, as these systems contribute to distinct phenotypes involved in intracellular survival.^36,54,74^ As such, we reasoned that CP-20’s dual inhibition of *agr* and ATP synthase may have an additive effect that would render *S. aureus* more susceptible to immune clearance than JE2 or either single mutant (JE2 *agrA::tn* and JE2 *atpA::tn*). To test this hypothesis, we infected immortalized murine macrophages with JE2 (untreated or CP-20-treated), JE2 *agrA::tn*, or JE2 *atpA::tn* and measured *S. aureus* survival after 5 h of infection. Consistent with previous findings,^36,54,74^ JE2 *agrA::tn* and JE2 *atpA::tn* exhibited moderately lower levels of intracellular survival (53% and 68%, respectively) relative to JE2 (80%). Excitingly, treatment with CP-20 caused JE2 killing to a far greater extent (24% survival) than untreated JE2, JE2 *agrA::tn*, or JE2 *atpA::tn* (Figure 4E) despite (again) not inhibiting *S. aureus* growth in the absence of macrophages (Figure S1).

To further probe the impact of simultaneous inhibition of *agr* and ATP synthase on immune clearance, we treated JE2, JE2 *agrA::tn*, and JE2 *atpA::tn* with the known *agr* inhibitor, AIP-III D4A, and evaluated intracellular survival (Figure 4F). We hypothesized that treating JE2 *atpA::tn* with AIP-III D4A would mimic simultaneous inhibition, thus rendering JE2 *atpA::tn* more susceptible to eradication than JE2 and JE2 *agrA::tn*. We observed no difference in survival between JE2 and JE2 *agrA::tn* when both were treated with AIP-III D4A (78% and 74% survival, respectively); this result is not surprising, as both have an inactive *agr* system and a functional ATP synthase under these conditions. However, and as anticipated, we observed a significant decrease in intracellular survival for JE2 *atpA::tn* treated with AIP-III D4A (41% survival) when compared to the other AIP-III D4A-treated conditions. These results provide further support that CP-20 inhibits both *agr* and ATP synthase and suggest that simultaneous inactivation of these two targets can render *S. aureus* more susceptible to immune clearance than inactivation of either target individually.

## Discussion

The ability of *S. aureus* to toggle between acute and chronic virulence phenotypes has contributed to its success as a human pathogen. Prior efforts to block these divergent phenotypes have revealed chemical inhibitors of either mode of pathogenesis. While effective in dampening phenotypes in certain infection contexts, these probes may not fully quench *S. aureus*’ virulence potential. In this study, we found a single compound—CP-20—that inhibits both *agr* and ATP synthase, enabling the inhibition of both acute and chronic virulence phenotypes. To our knowledge, CP-20 is the first dual inhibitor of *agr* and ATP synthase. Dual inhibition by a single molecule offers distinct advantages over cocktails of inhibitors of either target individually. For example, CP-20 has a similar potency toward both *agr* and ATP synthase, meaning that it can engage both targets, which potentially could confer therapeutic advantages over the use of two distinct inhibitors with different potencies and physicochemical properties. This feature should facilitate studies of the downstream compensatory responses of these two systems to concurrent inactivation, with temporal control, and provide a chemical means of linking central metabolism with virulence regulation.

Whether CP-20 inhibits ATP synthase via a direct or indirect mechanism is currently unknown. Activation of the SrrAB two-component system by a constitutively active response regulator has been shown to increase the expression of *atpB*, *atpC*, *atpD*, and *atpE*, suggesting that SrrAB may represent an indirect target through which ATP synthase can be regulated.^67^ We found that CP-20 is slightly activating in a fluorescent reporter that monitors SrrAB activity (Figure S7), indicating that its mechanism of ATP synthase inhibition is likely not dependent on SrrAB inhibition. Alternatively, the ability of CP-20 to interfere with the *S. aureus* membrane suggests that it may inhibit ATP synthase, at least in part, by remodeling the local lipid environment. CP-20’s synthetic tractability should enable the generation of reactive derivatives that could be used in labeling- or enrichment-based proteomics experiments to elucidate the specific biomolecular mechanism(s) by which CP-20 inhibits ATP synthase.

In the proteomics investigation described herein, we noted several proteins for which expression appeared to be regulated by CP-20, but that did not clearly align with *agr* or ATP synthase inactivity. These proteins hint at additional, currently unknown targets or possible downstream effects of CP-20’s membrane interference. Conversely, these proteins might be differentially regulated in response to the dual inhibition of *agr* and ATP synthase, perhaps signifying a “co-regulon” not observed when either system is selectively inactivated. Future experiments profiling the transcriptional changes present in both single and double mutants are necessary to exhaustively identify CP-20’s biomolecular targets and elucidate the impact of dual inhibition (or genetic inactivation) of *agr* and ATP synthase on virulence regulation in *S. aureus*.

Given *S. aureus*’ ability to switch between distinct lifestyles depending on the colonization or infection context, the inhibition of both “attack” and “hide” phenotypes by a single molecule offers significant potential for investigating and interfering with *S. aureus* pathogenicity in different settings. The *agr* system and ATP synthase are intriguing targets for such phenotypic modulation, as these systems are not required for growth in *S. aureus*. For example, the *agr* system positively regulates toxins and host evasion factors, enabling it to drive abscess formation and necrosis in SSTIs.^75^ A functional *agr* system has also been implicated in nasal and intestinal colonization by *S. aureus*.^28^ In these settings, *agr* inhibitors may effectively limit acute infection and colonization. In other contexts, *S. aureus* relies on a dysfunctional *agr* system to infect the host, making *agr* inhibitors less relevant to virulence control.^76,77^ One such context is in lung coinfections with *Pseudomonas aeruginosa* associated with cystic fibrosis, where *S. aureus* often takes on a “hide” lifestyle.^78^ In this disease setting, *S. aureus* tends to form antibiotic-resistant SCVs and modulates its staphyloxanthin production, which can protect both species from oxidative stress.^63,78^ Thus, ATP synthase inhibitors, which interfere with both SCV formation and staphyloxanthin production, have the potential to significantly limit *S. aureus*’ virulence and render both *S. aureus* and *P. aeruginosa* more susceptible to clearance by the host immune system in this chronic infection setting. We anticipate that the dual inhibition of *agr* and ATP synthase is likely to render *S. aureus* more susceptible to clearance in other infection models, including *in vivo* models of lung coinfections and diabetic wounds, wherein *S. aureus*’ dynamic phenotypes provide it with a fitness advantage.^62,63,78^

## Supplementary Material





## Notes

Reagents and materials; full method details; cell growth conditions; growth curves; additional biological assay and proteomics data (PDF)
MS proteomics data (XLSX)

## ⊥

PPD, 3230 Deming Way, Middleton, WI 53562, USA

## #

Department of Microbiology, Blavatnik Institute, Harvard Medical School, 25 Shattuck Street, Boston, MA 02115, USA

## ∇

Department of Molecular Biosciences, The University of Texas at Austin, 100 East 24^th^ Street, Austin, TX 78712, USA
