# Supplementary material for: A Small Molecule Dual Inhibitor of agr Quorum Sensing and ATP Synthase Blocks Acute and Chronic Virulence Phenotypes in Staphylococcus aureus
Source: ACS Infect Dis. 2026 Sep 23;12(10):3419–29. doi: 10.1021/acsinfecdis.6c00432 (PMC13660391; doi:10.1021/acsinfecdis.6c00432)
Supplement: Supplementary file 1 [file id6c00432_si_001.pdf]

SUPPORTING INFORMATION

A small molecule dual inhibitor of *agr* quorum sensing and ATP synthase blocks acute and chronic virulence phenotypes in *Staphylococcus aureus*

Troy D. Vulpis,[1] Jordan T. York,[1,†] A.P. Clay,[1] Thomas J. Polaske,[1,†] Samuel W. Markovich,[1] Kijeong Lee,[2,‡] Douglas H. Chang,[3,§] Sean P. Palecek,[3] David M. Lynn,[1,3] John-Demian Sauer,[2] Lloyd M. Smith,[1] and Helen E. Blackwell[1,*]

[1]Department of Chemistry, University of Wisconsin–Madison, 1101 University Ave., Madison, WI 53706, USA; [2]Department of Medical Microbiology and Immunology, University of Wisconsin–Madison, 1550 Linden Dr., Madison, WI 53706, USA; [3]Department of Chemical and Biological Engineering, University of Wisconsin−Madison, 1415 Engineering Dr., Madison, WI 53706, USA

**Correspondence:* blackwell@chem.wisc.edu

CONTENTS.

- Reagents and materials used in this study
- Cell growth conditions
- Method details
- **Figure S1.** CP-20 treatment does not significantly alter *S. aureus* growth
- **Table S1.** Minimum inhibitory concentration (MIC_{90}) values for selected antibiotics against *S. aureus* JE2 and *S. epidermidis* RP62A in the presence and absence of CP-20 or CP-20-I
- **Figure S2.** CP-20 alters protein abundance for *agr*- and ATP synthase-associated targets
- **Figure S3.** CP-20 can disrupt the *S. aureus* cell membrane
- **Figure S4.** CP-20 derivatives have varied cytotoxicity toward mammalian cell lines
- **Figure S5.** CP-20 selectively inhibits the growth of *S. aureus* small colony variants
- **Figure S6.** AIP-I inhibits *S. aureus* biofilm formation in an *agr*-dependent manner
- **Figure S7.** CP-20 weakly activates the SrrAB two-component system in *S. aureus*
- References

---

† J.T.Y. and T.J.P. — *Present Address: PPD, 3230 Deming Way, Middleton, WI 53562, USA*
‡ K.L. — *Present Address: Department of Microbiology, Blavatnik Institute, Harvard Medical School, 25 Shattuck Street, Boston, MA 02115, USA*
§ D.H.C. — *Present Address: Department of Molecular Biosciences, University of Texas at Austin, 100 East 24th Street, Austin, TX 78712, USA*

Reagents and materials used in this study.

REAGENT or RESOURCE	SOURCE	IDENTIFIER
Bacterial strains		
Staphylococcus aureus strain JE2	(Fey et al., 2013[1])	N/A
Staphylococcus aureus strain JE2 *agrA::tn*	(Fey et al., 2013[1])	N/A
Staphylococcus aureus strain JE2 *crtN::tn*	(Fey et al., 2013[1])	N/A
Staphylococcus aureus strain JE2 *atpA::tn*	(Fey et al., 2013[1])	N/A
Staphylococcus aureus strain JE2 *graR::tn*	(Fey et al., 2013[1])	N/A
Staphylococcus aureus strain JE2 *vraF::tn*	(Fey et al., 2013[1])	N/A
Staphylococcus aureus strain LAC13c	(Bosch et al., 2020[2])	N/A
Staphylococcus aureus strain LAC13c Δ*atpA*	(Bosch et al., 2020[2])	N/A
Staphylococcus aureus strain LAC13c Δ*atpA::atpA*	(Bosch et al., 2020[2])	N/A
Staphylococcus aureus strain MW2 7413	(Rapun-Araiz et al., 2020[3])	Contains plasmid pCN52::PmprF::gfp
Staphylococcus aureus strain MW2 7506	(Rapun-Araiz et al., 2020[3])	Contains plasmid pCN52::PMW1056::gfp
Staphylococcus aureus strain MW2 6178	(Rapun-Araiz et al., 2020[3])	Contains plasmid pCN52::Pqox::gfp
Staphylococcus epidermidis strain RP62A	ATCC	ATCC 35984
Cell lines		
Immortalized bone marrow-derived macrophages	Isolated from C57Bl6/J mice (Courtesy of Prof. J.D. Sauer)	N/A
Mouse fibroblast (NIH/3T3) cells	ATCC	CRL-1658
MDA-MB-231 triple-negative human breast cancer cells	ATCC	HTB-26
Human umbilical vein-endothelial cells	ATCC	PCS-100-010
Recombinant DNA		
pCN52::PmprF::gfp plasmid	(Rapun-Araiz et al., 2020[3])	N/A
pCN52::PMW1056::gfp plasmid	(Rapun-Araiz et al., 2020[3])	N/A
pCN52::Pqox::gfp plasmid	(Rapun-Araiz et al., 2020[3])	N/A
Biological samples		
Rabbit red blood cells, 10% suspension in Alsevers solution	Lampire Biological	Cat# 7246408
Fetal bovine serum	Corning	Cat# 35010CV
Chemical reagents		
Staphylococcus aureus AIP-I	(Tal-Gan et al. 2013[4])	N/A
Staphylococcus aureus AIP-III D4A	(Tal-Gan et al. 2013[4])	N/A
CP-20	(Polaske et al. 2025[5])	N/A
CP-20-I	(Polaske et al. 2025[5])	N/A
Dimethyl sulfoxide (DMSO)	Sigma-Aldrich	Cat# D5879
Methanol	Macron Fine Chemicals	Cat# 3016-16
Acetonitrile	Sigma-Aldrich	Cat# 34851
Erythromycin	Acros Organics	Cat# 227330050
Gentamicin sulfate	Sigma-Aldrich	Cat# G1264
Polymyxin B sulfate	Santa Cruz Biotechnology	Cat# sc-3544

Vancomycin	Santa Cruz Biotechnology	Cat# sc-204938A
Oxacillin	Research Products International	Cat# O21000
Linezolid	VWR International	Cat# 103837-270
Deoxycholic acid sodium salt	Fisher Bioreagents	Cat# BP349
Urea	Sigma-Aldrich	Cat# 51459
Sodium chloride (NaCl)	Sigma-Aldrich	Cat# 53014
Dithiothreitol (DTT)	Thermo Fisher	Cat# R0861
Tris (1 mM, pH 8)	Invitrogen	Cat# AM9856
Halt™ Protease and Phosphatase Inhibitor Cocktail (Halt PPI)	Thermo Fisher	Cat# 78440
Iodoacetamide	Sigma-Aldrich	Cat# I1149
Absolute ethanol	Thermo Fisher	Cat# T038181000
Ammonium bicarbonate	Sigma-Aldrich	Cat# 207861
Trypsin	Promega	Cat# V528A
Oligomycin A	Santa Cruz Biotechnology	Cat# sc-201551
SYTOX	Thermo Fisher	Cat# S7020
Triton X-100	Sigma-Aldrich	Cat# X100
2-heptyl-4-hydroxyquinoline-*N*-oxide (HQNO)	Ambeed	Cat# A1209614
Safranin O	Ward's Science	Cat# 470302-324
Acetic acid	Sigma-Aldrich	Cat# 695092
Dulbecco's phosphate-buffered saline	Gibco	Cat# 14190144
Sodium pyruvate (100 mM)	Gibco	Cat# 11360070
L-glutamine (200 mM)	Gibco	Cat# 25030081
β-mercaptoethanol	Gibco	Cat# 21985023
Penicillin-streptomycin (10,000 U/mL)	Thermo Fisher	Cat# 15140122
Trypsin-EDTA (0.25%) with phenol red	Thermo Fisher	Cat# 25200056
Commercial assays		
BacTiter-Glo	Promega	Cat# G8232
CellTiter-Glo	Promega	Cat# G7570
Pierce 660 Protein Quantification Assay	Thermo Fisher	Cat# 22662
Software		
Graphpad Prism, version 10.3.0	GraphPad Software	https://www.graphpad.com
MetaMorpheus, version 1.0.6	(Solntsev et al., 2018[6])	https://github.com/smith-chem-wisc/MetaMorpheus
Perseus, version 2.0.10.0	(Tyanova et al., 2016[7])	https://www.perseus-framework.org
ChemDraw Professional, version 23.1.1	Revvity Signals	https://revvitysignals.com/products/research/chemdraw
BioRender	BioRender	https://www.biorender.com
Other		
NutriSelect Basic tryptic soy broth	Sigma-Aldrich	Cat# T8907
Cation-adjusted Mueller Hinton broth	Sigma-Aldrich	Cat# 90922
Brain-heart infusion broth	Teknova	Cat# B9505

RPMI medium	Gibco	Cat# 11875093
Dulbecco's modified Eagle's medium	Gibco	Cat# 12491015
Endothelial cell growth medium-2 BulletKit	Lonza Bioscience	Cat# CC-3162
Sera-Mag™ carboxylate-modified magnetic beads	Cytiva	Cat# 29729997
Pierce™ C18 Pipette Tips, 100 μL	Thermo Fisher	Cat# 87782
C18 beads	Waters	Cat# 186004690

Cell growth conditions.

Bacterial cell growth conditions

All *S. aureus* strains were grown in NutriSelect Basic Tryptic Soy Broth (TSB), Brain-Heart Infusion Broth (BHI), or cation-adjusted Mueller-Hinton Broth (CA-MHB) as specified below. *S. aureus* transposon mutant strains (JE2 *agrA::tn*, JE2 *atpA::tn*, JE2 *crtN::tn*, JE2 *graR::tn*, and JE2 *vraF::tn*), LAC13c Δ*atpA*, and two-component system reporter strains (MW2 carrying pCN52::PmprF::gfp plasmid for GraSR, MW2 carrying pCN52::PMW1056::gfp plasmid for AgrCA, and MW2 carrying pCN52::Pqox::gfp plasmid for SrrBA) were supplemented with erythromycin (10 μg/mL).[1, 3] *S. epidermidis* RP62A was grown in CA-MHB. All bacteria were grown in a standard laboratory incubator at 37 °C with shaking at 200 rpm unless otherwise noted.

Mammalian cell growth conditions

Immortalized bone-marrow derived murine macrophages were grown in Roswell Park Memorial Institute (RPMI) medium supplemented with 10% (w/v) fetal bovine serum, 1% (w/v) pyruvate, 1% (w/v) glutamine, and 0.1% (w/v) β-mercaptoethanol (hereafter referred to as "Immortalized Macrophage Media"). Cells were grown without shaking in a 5% CO_2 incubator at 37 °C.

MDA-MB-231 triple-negative human breast cancer cells and 3T3 mouse fibroblast (NIH/3T3) cells were grown in Dulbecco's modified Eagle's medium supplemented with 10% (w/v) fetal bovine serum and 100 units/mL penicillin-streptomycin. Human umbilical vein-endothelial cells (HUVECs) were grown in endothelial cell growth medium-2 (EGM-2). Cells were grown without shaking in a 5% CO_2 incubator at 37 °C.

Method details.

Compound handling and preparation

S. aureus AIP-I and *S. aureus* AIP-III D4A were synthesized according to previously reported methods and acquired from in-house stocks.[4] Briefly, linear peptides were synthesized via Fmoc/tBu solid-phase peptide synthesis using Dawson Dbz AM resin, followed by solution-phase macrocyclization and HPLC purification. CP-20 and CP-20-I were synthesized according to previously reported methods and acquired from in-house stocks.[5] Briefly, CP-20 and its derivatives were synthesized via a thiazole condensation followed by successive amide and urea formations. All synthesized and purchased compounds were prepared as stock solutions in DMSO and stored at -20 °C. In all experiments, CP-20 was diluted in media or buffer and

administered at concentrations required for complete inhibition of ATP synthase in cell-based assays (≥ 10 μM) to measure ATP synthase-dependent phenotypes or in dose-response format (see below for assay-specific compound handling protocols).

Instrumentation

Bacterial cultures were grown in a standard laboratory incubator heated to 37 °C with shaking at 200 rpm unless otherwise specified. UV-Vis absorbance measurements were taken using a NanoDrop 2000c spectrophotometer (Thermo Fisher). All experiments requiring a plate reader were conducted using a Biotek Synergy 2 plate reader running Gen 5 software (version 1.05) or a Tecan Infinite 200 Pro plate reader running i-control software (version 3.40). Cell lysis for proteomics experiments was performed using a Model 505 Sonic Dismembrator (Fisherbrand). Proteomics samples were concentrated using a SpeedVac Concentrator (Thermo Fisher). Mass spectrometry (MS) data for proteomics experiments were collected on an Orbitrap Fusion Lumos mass spectrometer equipped with a Neo Vanquish ultra-high-pressure liquid chromatography (UPLC) system (Thermo Fisher). All other MS data were collected on a Q-Exactive FOCUS ESI-Q-Orbitrap mass spectrometer equipped with an UltiMate 3000 HPLC and a diode array detector (Thermo Fisher).

S. aureus growth curves

Overnight cultures of *S. aureus* JE2 were grown from a single colony in TSB or BHI (3 mL) for 16 hr at 37 °C with shaking at 200 rpm. Overnight cultures were diluted 1:200 in fresh TSB or BHI. Aliquots (2 μL) of CP-20 (derived from a stock solution in DMSO) or DMSO (vehicle control) were added to the wells of a clear polystyrene 96-well microtiter plate (Costar). To each well containing CP-20 or DMSO, aliquots (198 μL) of diluted culture were added. CP-20 was tested at final concentrations of 1 μM, 10 μM, and 25 μM. Sterile TSB or BHI were included as media sterility controls. Plates were incubated for 16 hr at 37 °C on “fast shaking” mode in a plate reader, and OD_{600} measurements were taken every 10 min. Three biological replicates were collected for each experimental condition.

Staphyloxanthin quantification assay

Staphyloxanthin was quantified as previously described,[8] with minor modifications. Overnight cultures of selected *S. aureus* strains were grown from a single colony in TSB (3 mL) for 16 hr at 37 °C with shaking at 200 rpm. Overnight cultures were diluted 1:1000 in fresh TSB (3 mL) and transferred to sterile test tubes (16 mL volume). To each test tube containing diluted culture, aliquots (30 μL) of compound (prepared from stock solutions in DMSO) or DMSO (vehicle control) were added. Each condition contained 1% (v/v) DMSO. Compound- or vehicle-treated subcultures were incubated for 24 hr at 37 °C with shaking at 200 rpm. Aliquots (200 μL) of these cultures were removed, and OD_{600} measurements were taken using a plate reader. A second aliquot (1 mL) of culture was then removed and centrifuged at 13,000 rpm for 1 min at room temperature and supernatant was discarded. Pelleted cells were resuspended in methanol (400 μL) and incubated at 50 °C for 20 min to extract staphyloxanthin from the cell pellet. Cell suspensions in methanol were centrifuged at 13,000 rpm for 2 min at room temperature to pellet cells. Supernatant (2 x 200 μL aliquots) containing extracted staphyloxanthin were removed and their absorbance at 465 nm (A465) was measured using a plate reader. Absorbance measurements were corrected to methanol background (i.e., methanol A465 set to 0) and normalized to OD_{600} and vehicle control (i.e., vehicle A465 set to 1). Dose-response curves,

maximum inhibition values, and potency values were generated by four-parameter non-linear regression analysis ([Inhibitor] vs. response) using GraphPad Prism software. At least three biological replicates were collected for each experimental condition.

The same protocol was followed for experiments in which complete absorbance spectra for staphyloxanthin were collected, with minor modifications. When collecting the methanol extract, a single aliquot (400 µL) was collected, diluted to 1 mL in water, and transferred to a cuvette. Absorbance (300-800 nm) of diluted extract then was measured using a NanoDrop spectrophotometer.

Minimum inhibitory concentration determination

Minimum inhibitory concentration assays were performed as previously described with some minor modifications.[9] Overnight cultures of *S. aureus* JE2 or associated transposon mutants (*agrA::tn* or *atpA::tn*) were grown from a single colony in CAMHB for 18-20 hr at 37 °C with shaking at 200 rpm. Overnight cultures were diluted in fresh CAMHB to approximately 10^6 CFU/mL. In a white tissue culture-treated 96-well microtiter plate (Costar), two-fold broth microdilutions of selected antibiotics were prepared. DMSO or sterile water were included as vehicle controls. To all wells containing antibiotic, aliquots (1 µL) of CP-20 (final concentration of 25 µM) or DMSO (vehicle control) were added. CP-20 was administered at 25 µM because this concentration was sufficient to maximally inhibit ATP synthesis in cell-based assays. Separate wells containing CP-20 or DMSO alone were included as antibiotic-free growth controls. An aliquot (50 µL) of diluted culture was added to all wells containing antibiotic or vehicle control. An aliquot (100 µL) of CAMHB was used as a media sterility control. Plates were incubated at 37 °C without shaking for 20-24 hr depending on the antibiotic tested (in accordance with CLSI guidelines for MIC determination).[9] OD_{600} values were measured using a plate reader, corrected to media background (i.e., media OD_{600} set to 0%), and normalized to vehicle control (i.e., vehicle OD_{600} set to 100%). The MIC_{90} concentration was determined to be the most dilute concentration of antibiotic that inhibited growth by ≥90% relative to the respective vehicle control. Three biological replicates were collected for each experimental condition.

Bottom-up proteomics experiments

Preparation of bottom-up proteomics samples. Overnight cultures of *S. aureus* JE2, JE2 *agrA::tn*, and JE2 *atpA::tn* were grown from a single colony in TSB (3 mL) for 16 hr at 37 °C with shaking at 200 rpm. Overnight cultures were diluted 1:200 in fresh TSB, and aliquots (3 mL) of each diluted culture were transferred to sterile test tubes (16 mL volume). To each test tube containing diluted culture, aliquots (3 µL) of CP-20 (from a 10 mM stock solution in DMSO) or DMSO (vehicle control) were added. Treated samples were incubated for 6 hr at 37 °C with shaking at 200 rpm. Following this incubation, aliquots (1 mL) of each sample were removed and centrifuged at 13,000 rpm for 5 min. Pelleted cells (approximately 10^{10} CFU) were isolated and washed 3x with PBS before being treated with lysis buffer containing 2% Triton X-100, 2% deoxycholic acid, 8M urea, 500 mM NaCl, 5 mM DTT, 100 mM Tris pH 8, and 1x Halt™ Protease and Phosphatase Inhibitor Cocktail. This suspension was incubated for 18 hr at 4 °C on a nutator. Samples were lysed by sonication for 30 sec on, 30 sec off for 3 cycles. Lysates were clarified by centrifugation at 10,000x*g* for 15 min at 4 °C. Samples were incubated for 30 min at 37 °C to ensure complete reduction of disulfides by DTT, then alkylated with 30 mM iodoacetamide for 30 min in the dark. Samples were prepared for mass spectrometry using the SP3 solid-phase purification method as previously described.[10] Briefly, Sera-Mag™ carboxylate-modified magnetic beads (500 µg) were added to each 200 µL sample containing15 µg of

protein as determined by Pierce 660 Protein Quantification Assay, followed by an equal volume of molecular biology grade absolute ethanol. Samples were mixed at 900 rpm for 5 min at room temperature, followed by a brief benchtop centrifugation. Samples were incubated on a magnetic stand for 2 min, after which the supernatant was removed. Beads were washed by resuspension in 80% ethanol (500 µL), placed on a magnetic stand for 2 min, and the supernatant was removed. This wash procedure was repeated 3x for a total of four washes. Beads were resuspended in 50 mM ammonium bicarbonate pH 7.5 (200 µL) with trypsin (1 µg) and nutated for 2 hr at 47 °C. Samples then were centrifuged at 20,000xg for 1 min at room temperature and carefully transferred to a magnetic stand to not disturb the pelleted beads. The supernatant, containing tryptic peptides, was transferred to a new tube. Samples were immediately desalted using a Pierce™ C18 solid-phase extraction pipette tip (100 µL) according to manufacturer's protocols. Desalted samples were dried using a SpeedVac concentrator and reconstituted in 95:5 H_2O:Acetonitrile (ACN), 0.1% formic acid for mass spectrometric analysis.

High-performance liquid chromatography-tandem mass spectrometry (HPLC-MS/MS) analysis for proteomics experiments. Desalted tryptic peptide samples were analyzed via UPLC-MS/MS. Two buffers were used in all experiments: buffer A = H_2O, 0.1% formic acid and buffer B = 80% acetonitrile: 20% H_2O, 0.1% formic acid. Peptides were loaded in buffer A at a pressure of 300 Bar onto a 20 cm long fused silica capillary nano-column packed with C18 beads (1.7 µm-diameter, 130 Angstrom pore size, Waters). Peptides eluted over 120 min at a flow rate of 350 nL/min with the following gradient: time 1 min, 5% buffer B; time 1-52 min, 30% buffer B; time 80 min, 42% buffer B; time 90 min, 55% buffer B; time 95 to 100 min, 85% buffer B; time 101 to 120 min, equilibrate at 0% buffer B. The nano-column was held at 60 °C using a column heater (constructed in-house).[11]

The nanospray source voltage was set to 2,200 V. Full-mass profile scans were performed in the orbitrap between 375-1,500 m/z at a resolution of 120,000, followed by MS/MS HCD scans in the orbitrap of the highest intensity parent ions in a 3 sec cycle time at 30% relative collision energy and 15,000 resolution, with a 2.5 m/z isolation window. Charge states of 2-6 were included, and dynamic exclusion was enabled with a repeat count of one over a duration of 30 sec and a 10-ppm exclusion width (both low and high). The automatic gain control target was set to "standard", maximum inject time was set to "auto", and a 1 µscan was collected for the MS/MS orbitrap HCD scans. Three biological replicates were collected for each experimental condition.

LC-MS/MS data searches and differential abundance analysis for proteomics experiments. Spectral files were analyzed with the free and open-source search software program MetaMorpheus (version 1.0.6, https://www.github.com/smith-chem-wisc/MetaMorpheus). The National Center for Biotechnology Information database for *S. aureus* (Taxonomy ID 451515) was used as the reference proteome, along with the internal common contaminants list in MetaMorpheus. Files were calibrated, averaged, and subject to global posttranslational modification discovery (GPTMD). Searches were performed with standard settings, including label-free quantification, match between runs, and normalization of quantification results. A 1% false discovery rate for both peptides and proteins was applied. To determine differentially abundant proteins, pairwise comparisons were made between protein abundances in different sample types by applying a Student's *t*-test using Perseus software.[7] All processed proteomics data with calculated differential abundances can be found in the Supplemental Data File (.xlsx).

Two-component system reporter assays

Overnight cultures of *S. aureus* 7506 (AgrCA), 7514 (GraSR), and 6178 (SrrBA) two-component system reporter strains were grown from single colonies in TSB (3 mL) for 24 hr at 37 °C with shaking at 200 rpm. Overnight cultures were diluted 1:100 in fresh TSB (3 mL) and transferred to sterile test tubes (16 mL volume). To each test tube containing diluted culture, aliquots (3 µL) of compound (prepared from stock solutions in DMSO) or DMSO (vehicle control) were added. Treated cultures were incubated for an additional 24 hr at 37 °C with shaking at 200 rpm. Following this incubation, an aliquot (1 mL) was removed from each culture and centrifuged at 13,000 rpm for 2 min. Supernatant was discarded, and pelleted cells were washed once with PBS (1 mL). Resuspended cells were then centrifuged at 13,000 rpm for 2 min, supernatant was discarded, and cell pellets were resuspended in PBS (100 µL). Concentrated cell suspensions were transferred to a black polystyrene 96-well microtiter plate (Costar). Fluorescence and OD_{600} values were measured using a plate reader, and fluorescence measurements were corrected to media background fluorescence (i.e., media fluorescence set to 0%) and normalized to OD_{600} and vehicle control (i.e., vehicle fluorescence set to 100%). Three biological replicates were collected for each experimental condition.

Intracellular ATP quantification

ATP measurements were performed as previously described with minor modifications.[12] Overnight cultures of selected *S. aureus* strains were grown from a single colony in CAMHB (3 mL) for 16 hr at 37 °C with shaking at 200 rpm. After overnight growth, cells were pelleted by centrifugation at 13,000 rpm for 2 min. Cells were then washed 1x with PBS before being diluted to an OD_{600} of 0.15 in fresh CAMHB. In parallel, either DMSO (1 µL) or compound (1 µL) were added to the wells of a white polystyrene 96-well microtiter plate (Costar). To each well containing DMSO or compound, diluted cell culture (99 µL) was added. Plates were incubated for 1 hr at 37 °C with shaking at 700 rpm. After incubation, OD_{600} was measured using a plate reader. Aliquots (100 µL per well) of BacTiter-Glo reagent were then added to each well containing cell culture, and the plate was incubated for 5 min in the dark. Luminescence was measured using a plate reader and normalized to OD_{600} to determine an approximate concentration of ATP adjusted for cell growth. Dose-response curves, maximum inhibition values, and potency values were generated via four-parameter non-linear regression analyses ([Inhibitor] vs. response) using GraphPad Prism software. Three biological replicates were collected for each experimental condition.

SYTOX membrane disruption assays

Overnight cultures of *S. aureus* JE2 were grown from a single colony in TSB (3 mL) for 16 hr at 37 °C with shaking at 200 rpm. Overnight cultures were diluted 1:100 in fresh TSB (3 mL) and incubated for an additional 6 hr at 37 °C with shaking at 200 rpm. Following this incubation, aliquots (1 mL) of each sample were removed, and centrifuged at 13,000 rpm for 5 min. Pelleted cells were washed 3x with sterile PBS before being diluted 1:10 in sterile PBS. In parallel, aliquots (1 µL) of DMSO, CP-20, CP-20-I, or Triton X-100 were added to the wells of a white polystyrene 96-well microtiter plate (Costar). CP-20 and its derivatives were added to a final concentration of 25 µM. Triton X-100 was added to a final concentration of 0.1% (v/v). Next, an aliquot (1 µL) of SYTOX, derived from a 1 mM stock solution in DMSO, was added to each well containing DMSO or compound (final concentration of 10 µM). To each well containing compound and SYTOX, an aliquot (98 µL) of diluted cell culture in PBS was added and gently mixed via aspiration. Plates were incubated statically in a plate reader, and fluorescence and

OD_{600} were measured every 5 min for 1 hr. Three biological replicates were collected for each experimental condition.

Mammalian cell toxicity assays

Mouse fibroblast (NIH/3T3) cells, MDA-MB-231 triple-negative human breast cancer cells, and human umbilical vein-endothelial cells were grown as described above (see *Mammalian cell growth conditions* for growth media specifications for each cell line). Cells were passaged upon 70–80% confluency by gently washing the cell layer with PBS and then detaching them from the bottom of the wells with a 0.25% trypsin–EDTA solution. Detached cells were seeded onto tissue-culture treated polystyrene 96-well microtiter plates (Costar) at a density of 2.5 x 10^5 cells/mL in growth media (100 µL per well). Plated cells were incubated for 24 hr at 37 °C in an incubator supplemented with 5% CO_2. Following incubation, three-fold serial dilutions of CP-20 (derived from a stock solution in DMSO) were diluted in growth media (100 µL) and added to each well. DMSO was included as a vehicle control. Compound- or DMSO-treated cells were then incubated for an additional 48 hr at 37 °C in an incubator supplemented with 5% CO_2. Mammalian cell viability was characterized using a CellTiter-Glo metabolic activity assay. The assay was performed according to the manufacturer's recommendations by adding the CellTiter-Glo reagent (100 µL) to each well containing cells. Next, an aliquot (100 µL) of supernatant in each well was transferred into an opaque-bottomed white 96-well microtiter plate (Costar), and the luminescence signal was quantified using a plate reader. Luminescence measurements were corrected to media background luminescence (i.e., media luminescence set to 0%) and normalized to vehicle control (i.e., vehicle luminescence set to 100%). Three biological replicates were collected for each experimental condition.

Red blood cell hemolysis assays

Overnight cultures of selected *S. aureus* strains were grown from single colonies in TSB (3 mL) for 16 hr at 37 °C with shaking at 200 rpm. Overnight cultures were diluted 1:200 in fresh TSB, and aliquots (3 mL) of each diluted culture were transferred to sterile test tubes (16 mL volume). To each test tube containing diluted culture, aliquots (3 µL) of compound (prepared from stock solutions in DMSO) or DMSO (vehicle control) were added. Compound-treated samples were incubated for an additional 6 hr at 37 °C with shaking at 200 rpm. Following incubation, cells were pelleted by centrifugation at 13,000 rpm for 5 min. Supernatant was removed and filtered using a 0.2 µm syringe filter. Filtered supernatant was serially diluted two-fold in sterile water. Aliquots of supernatant dilution series (50 µL) were plated in a round-bottom polystyrene 96-well microtiter plate (Costar). A solution of 10% Triton X-100 (50 µL/well) served as a positive control for hemolysis. Sterile TSB (50 µL/well) served as a negative control for hemolysis. To each well containing cell supernatant, Triton, or TSB, aliquots of a 2.5% (v/v) suspension of rabbit red blood cells (50 µL/well) were added. Wells were mixed by gently aspirating with a multi-channel pipette. Plates were then incubated for 1 hr at 37 °C without shaking before being centrifuged at 300xg for 5 min to pellet cells. Supernatant (75 µL/well) was then removed and transferred to a clear polystyrene 96-well microtiter plate (Costar). Absorbance was measured at 425 nm (A425) to quantify relative hemolysis using a plate reader. Absorbance measurements were corrected to media background (i.e., TSB A425 set to 0% hemolysis) and normalized to OD_{600} and Triton X-100 (i.e., Triton X-100 A425 set to 100% hemolysis). Dose-response curves were generated via four-parameter non-linear regression analysis ([Inhibitor] vs. response) using GraphPad Prism software. Three biological replicates were collected for each experimental condition.

LC-MS δ-toxin quantification

Overnight cultures of selected *S. aureus* strains were grown from single colonies in TSB (3 mL) for 16 hr at 37 °C with shaking at 200 rpm. After overnight incubation, each culture was diluted 1:200 in fresh TSB, and aliquots (3 mL) of each diluted culture were transferred to sterile test tubes (16 mL volume). To each test tube containing diluted culture, aliquots (3 μL) of compound (prepared from stock solutions in DMSO) or DMSO (vehicle control) were added. Compound-treated samples were incubated for an additional 6 hr at 37 °C with shaking at 200 rpm. Following incubation, aliquots (1 mL) of each sample were removed, centrifuged at 13,000 rpm for 5 min. Supernatant was collected and filtered with a 0.2 μm syringe filter. Filtered supernatant (5 μL) was directly injected on a HPLC/MS instrument equipped with an Agilent PLRP-S 300A column (50 mm x 2.1 mm) using the following HPLC method: flow rate = 300 μL/min; buffer A = H_2O, 0.1% formic acid and buffer B = acetonitrile, 0.1% formic acid; gradient = time 0-2.5 min, hold at 0% buffer B; time 12.1 min, 60% buffer B; time 12.6, 90% buffer B; time 12.6-14.1 min, hold at 90% buffer B; time 14.6, 0% buffer B; time 14.6-15.6 min, equilibrate at 0% buffer B. The first 5 min of eluent were flushed to waste using a divert valve to prevent salt build-up. Peaks corresponding to δ-toxin were identified in the chromatograms and quantified based on MS^1 extracted ion chromatograms with a mass tolerance of 5 ppm. All δ-toxin integrated peaks were normalized to the TIC to account for slight variability in ionization efficiency. Three biological replicates were collected for each experimental condition.

Small colony variant susceptibility assays

Overnight cultures of *S. aureus* JE2 were grown from a single colony in BHI (3 mL) for 16 hr at 37 °C with shaking at 200 rpm. Aliquots (2 μL) of HQNO (from a 10 mg/mL stock solution in DMSO) were plated in a clear polystyrene 96-well microtiter plate (Costar) to induce small colony variant (SCV) formation.[13] DMSO (2 μL) was plated in parallel as a control for normal colony variant (NCV) formation. To each well containing HQNO or DMSO, aliquots (2 μL) of CP-20 (two-fold dilution series from a stock concentration of 5 mM in DMSO) were plated. DMSO (2 μL) was plated as a vehicle control. Each well contained a total volume of 4 μL DMSO (2%). To each well containing compound, diluted overnight culture was plated (196 μL). BHI (200 μL) was plated in parallel as a sterility control. The plate was incubated for 24 hr at 37 °C without shaking. Following this incubation period, OD_{600} was measured using a plate reader. Three biological replicates were collected for each experimental condition.

Biofilm growth and quantification

Overnight cultures of selected *S. aureus* strains were grown from single colonies in TSB (3 mL) for 16 hr at 37 °C with shaking at 200 rpm. In parallel, aliquots (5 μL) of compounds (prepared from stock solutions in DMSO) or DMSO (vehicle control) were plated in a clear, flat-bottom, tissue culture-treated polystyrene 24-well microtiter plate (Costar). Overnight cultures were then diluted 1:500 and supplemented with 1% (w/v) glucose. Diluted culture (495 μL) was added to the wells containing compound or DMSO. TSB (500 μL) was included as a sterility control. Plates were incubated for 24 hr at 37 °C without shaking.

The amount of biofilm formed in the wells was measured using a safranin staining method.[14, 15] Following incubation, the planktonic cultures were removed via gentle aspiration. The wells were gently washed with PBS (500 μL) and fixed in an oven for 1 hr at 50 °C. Adherent biofilm in the wells was incubated in a 0.1% aq. safranin solution (500 μL) for 25 min at room temperature. Non-adherent stain was removed by washing with water (1 mL). To solubilize the

safranin stain, a 30% aq. solution of acetic acid (500 μL) was added to each well. The plates were then incubated for 60 min at room temperature with rocking at 60 rpm. Next, aliquots (2 x 200 μL) of the solubilized dye were diluted eightfold and transferred to a clear polystyrene 96-well microtiter plate (Corning). Safranin was quantified by measuring absorbance at 535 nm (A535) using a plate reader. Absorbance measurements were corrected to media background (i.e., TSB A535 set to 0% biofilm). Three biological replicates were collected for each experimental condition.

Intracellular S. aureus survival assays

Immortalized mouse bone marrow-derived macrophages were seeded into the wells of a tissue culture-treated polystyrene 24-well microtiter plate (Costar) at approximately 2.5 x 10^5 cells per well. Macrophages were incubated in immortalized macrophage media (IMM) for approximately 18 hr at 37 °C in an incubator supplemented with 5% CO_2. This growth period allowed the macrophages to adhere to the plate and double to the target density of 5 x 10^5 cells per well. In parallel, cultures of selected *S. aureus* strains were grown in TSB (3 mL) for 16 hr at 37 °C with shaking at 200 rpm. To each of these cultures, aliquots (30 μL) of compound (prepared from stock solutions in DMSO) or DMSO (vehicle control) were added. Following overnight incubation, *S. aureus* cultures were washed with PBS and resuspended in sterile IMM. In parallel, macrophages were washed with PBS and resuspended in sterile IMM in the microtiter plate. Compound- or DMSO-treated *S. aureus* cultures were added to wells containing macrophages at a multiplicity of infection = 10 (macrophages at 5 x 10^5 cells/well, *S. aureus* at 5 x 10^6 CFU/well). Each well contained a total volume of 500 μL IMM. The infected macrophages were centrifuged at 500xg for 10 min to initiate contact. Infected macrophages were then incubated for 10 min at 37 °C in an incubator supplemented with 5% CO_2. Next, the infected macrophages were washed with PBS, and the media was replaced with fresh IMM containing gentamicin (50 μg/mL) to kill extracellular *S. aureus*. Half of the infected macrophages were incubated for an additional 10 min (time point #1, total t = 0.5 hr), while the other half of the infected macrophages were incubated for an additional 4.5 hr (time point #2, total t = 5 hr). At each time point (t = 0.5 hr and t = 5 hr), cells were washed with PBS and lysed with a solution of 1% aq. saponin (1 mL per well). *S. aureus*-containing macrophage lysate was serially diluted and plated on tryptic soy agar plates, which were incubated overnight at 37 °C prior to *S. aureus* CFU enumeration. For each condition, the t = 0.5 hr time point was normalized to 100% (i.e., CFU at the start of infection set to 100%) to evaluate relative intracellular killing at the t = 5 hr time point. At least six biological replicates were collected for each experimental condition.

Supplemental figures and table.

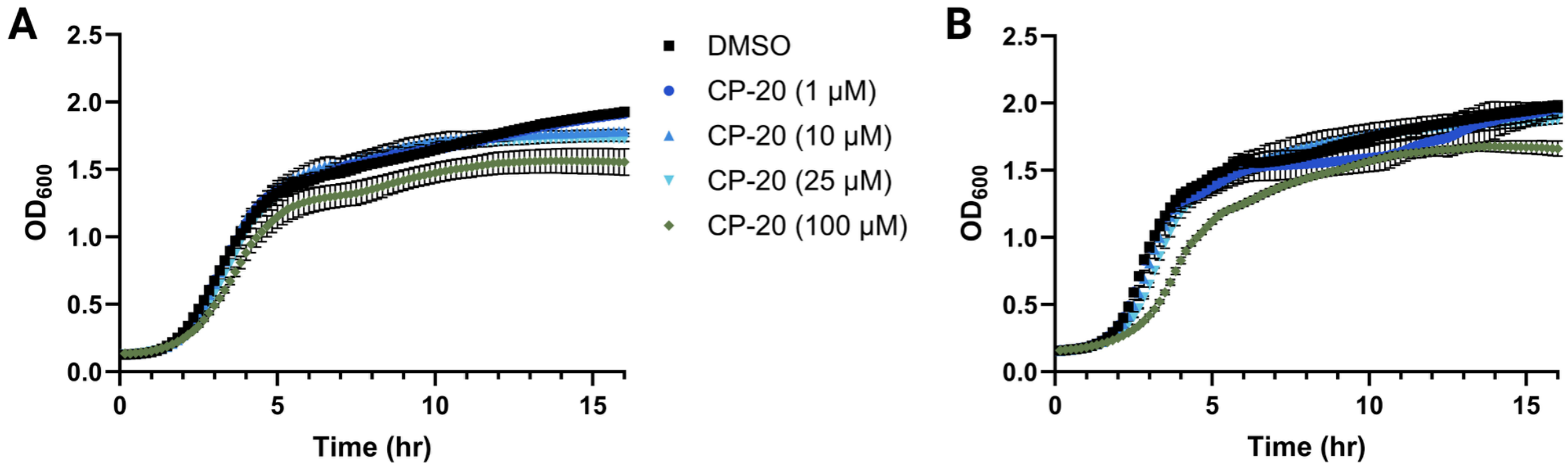

Figure S1. CP-20 treatment does not significantly alter *S. aureus* growth. Growth curves for *S. aureus* JE2 treated with CP-20 at various concentrations or DMSO (vehicle control) when grown in **(A)** TSB or **(B)** BHI. Cells were incubated in a plate reader at 37 °C on fast shaking mode. OD_{600} measurements were taken every 10 min for 16 hr. Data are shown as mean ± SEM for three biological replicates.

Table S1. Minimum inhibitory concentration (MIC_{90}) values for selected antibiotics against *S. aureus* JE2 and *S. epidermidis* RP62A in the presence and absence of CP-20 or CP-20-I (both at 25 μM).

**Strain ▸**	***S. aureus* JE2**	***S. aureus* JE2 + CP-20-I**	***S. epidermidis* RP62A**	***S. epidermidis* RP62A + CP-20**
**Antibiotic ▾**	MIC_{90} (μg/mL)	MIC_{90} (μg/mL)	MIC_{90} (μg/mL)	MIC_{90} (μg/mL)
**Polymyxin B**	128	16–32	128	32
**Gentamicin**	1	0.25	128[a]	32[b]

[a] MIC_{75} was calculated because gentamicin did not reduce cell growth by ≥90% under these conditions.
[b] Treatment with CP-20 (25 μM) restored cell killing activity to ≥90% (MIC_{90} was calculated for CP-20-treated cells) and shifted the MIC four-fold compared to DMSO treatment.

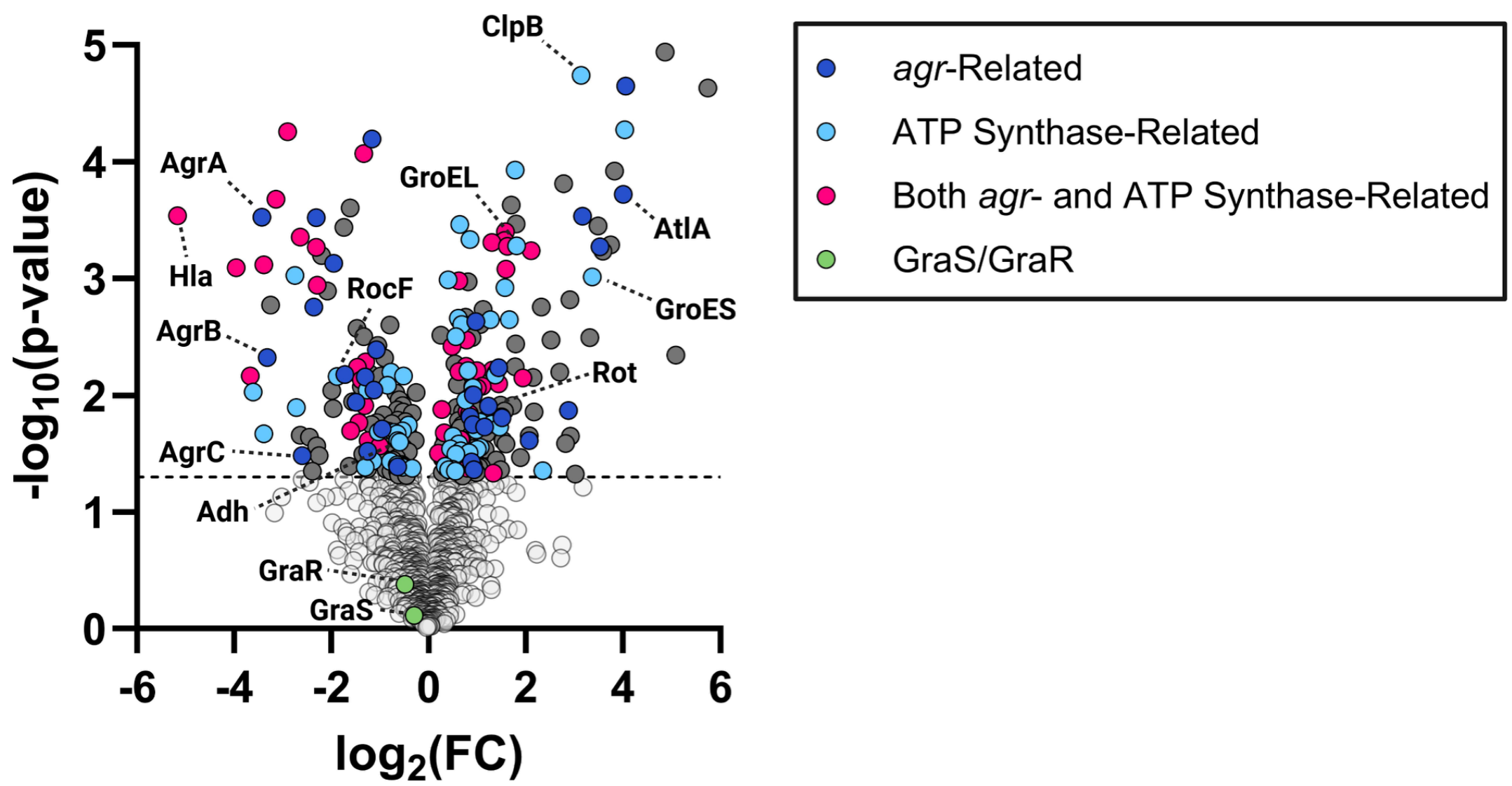

Figure S2. CP-20 alters protein abundance for *agr*- and ATP synthase-associated targets. Volcano plot depicting changes in protein abundance for *S. aureus* JE2 following treatment with CP-20 (10 µM) or DMSO (vehicle control). Colored circles represent proteins that exhibit the same directional change in abundance between CP-20-treated cells and *S. aureus* strains with single mutations to *agr* (dark blue), ATP synthase (light blue), or both *agr* and ATP synthase (pink) (derived from this study and previous reports[2, 16]). Green circles indicate the GraSR two-component system. Relative protein abundance was determined via label-free quantification and is shown as the fold change (FC) in protein abundance for CP-20-treated cells relative to DMSO control. A positive FC value indicates higher abundance in CP-20-treated cells than DMSO-treated cells and *vice versa* for a negative FC value. *p*-values were determined using a paired student's t-test and reflect the statistical confidence in the calculated differences in protein abundance between the two treatment conditions. Points above the dashed line represent proteins for which abundance changed with a $p < 0.05$. Data are shown as the mean of three biological replicates.

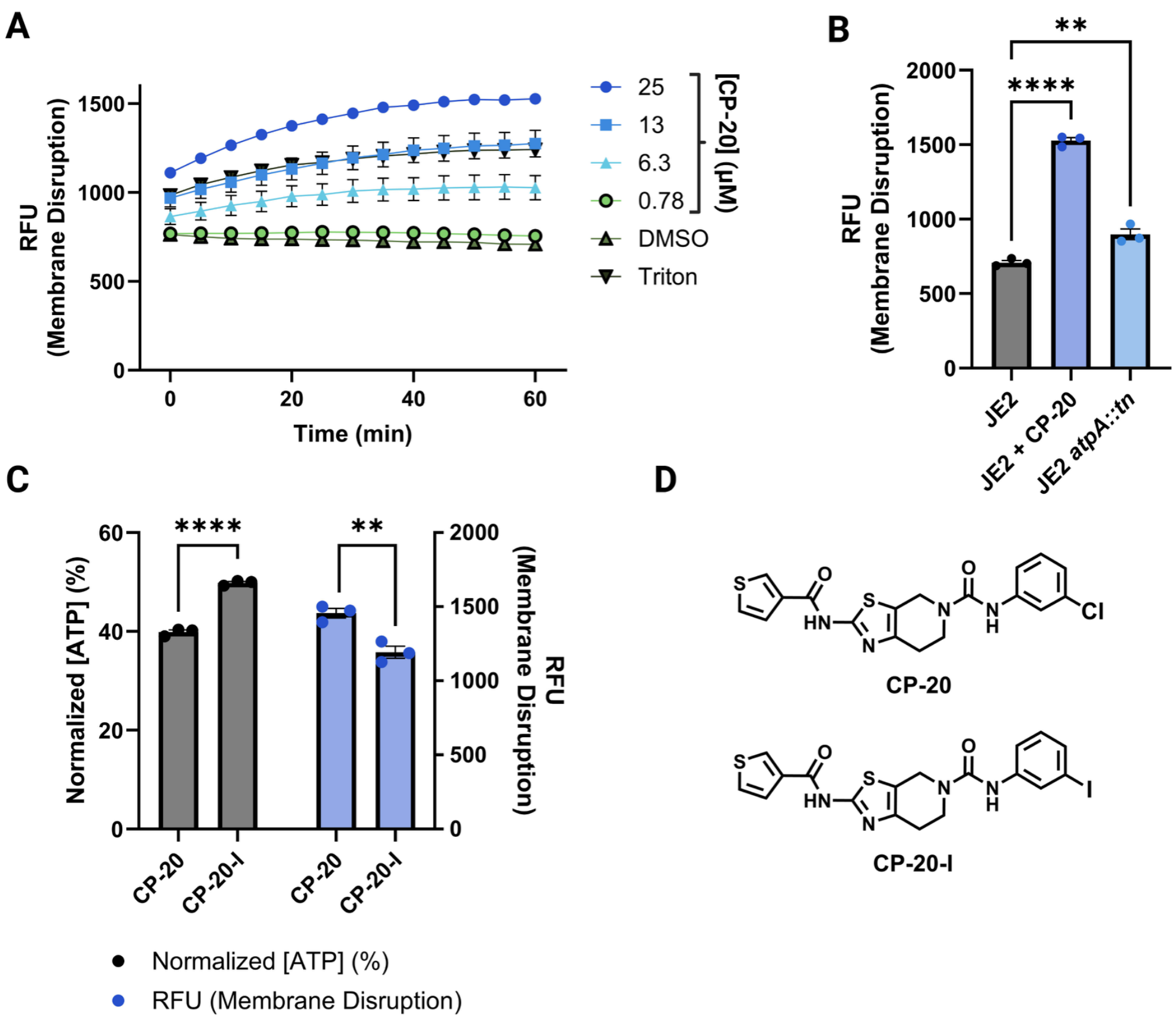

Figure S3. CP-20 can disrupt the *S. aureus* cell membrane. **(A)** Time-course SYTOX membrane disruption data for *S. aureus* JE2 treated with DMSO, CP-20 (varied concentrations in µM), or Triton X-100 (0.1% v/v). **(B)** Bar plot comparing the membrane disruption observed from JE2 treated with DMSO or CP-20 (25 µM) and JE2 *atpA::tn* treated with DMSO. Membrane disruption values were determined using the SYTOX membrane disruption assay and are given as raw fluorescence unit (RFU) measurements taken at the 60-minute time point. **(C)** Bar plot comparing the effects of CP-20 and CP-20-I treatment (both at 25 µM) on ATP concentration (determined using a BacTiter-Glo-based ATP quantification assay) and membrane disruption (determined using the SYTOX based membrane disruption assay). ATP concentrations are normalized to DMSO vehicle control (set to 100%). Membrane disruption values are given as RFU measurements taken at the 60-minute time point. **(D)** Chemical structures of CP-20 and CP-20-I. Data are shown as mean ± SEM for three biological replicates. ** $\alpha < 0.01$, **** $\alpha < 0.0001$ by an ordinary one-way ANOVA with Dunnett's multiple comparisons test (panel B). ** $p < 0.01$, **** $p < 0.0001$ by a Student's t-test (panel C).

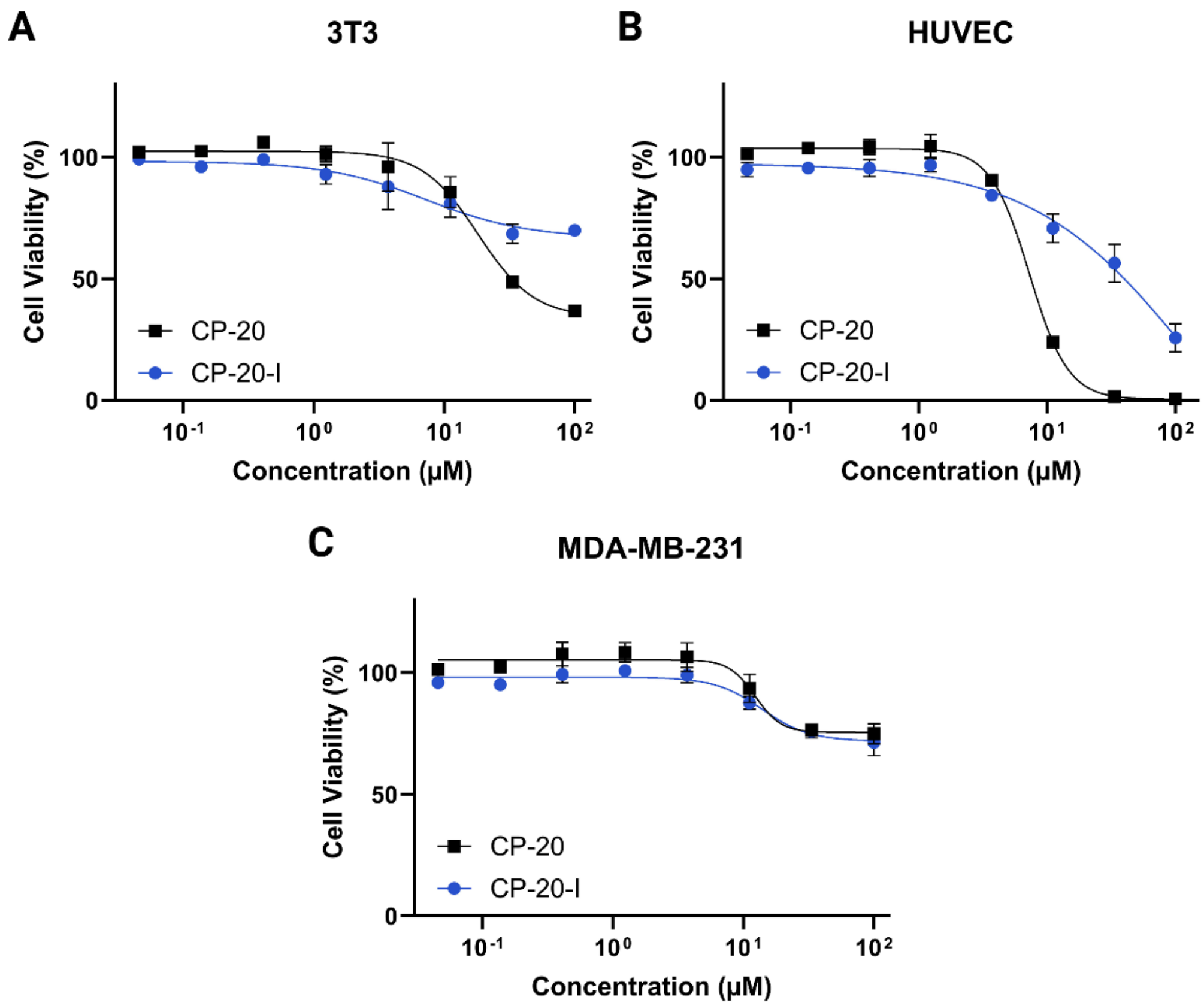

Figure S4. CP-20 derivatives have varied cytotoxicity toward mammalian cell lines. Cell viability data for **(A)** 3T3, **(B)** HUVEC, and **(C)** MDA-MB-231 cell lines treated with CP-20 or CP-20-I over a range of concentrations. Cell viability was measured using CellTiter-Glo following manufacturer instructions. Data are shown as mean ± SEM for three biological replicates.

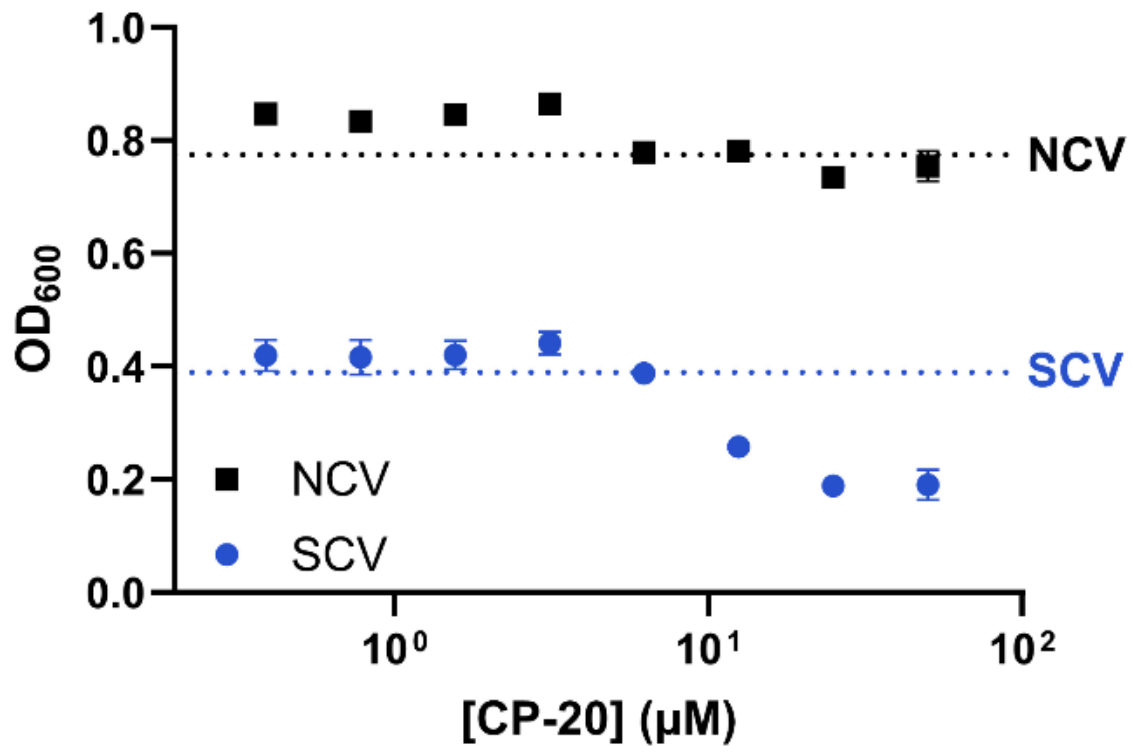

Figure S5. CP-20 selectively inhibits the growth of *S. aureus* small colony variants. Cell growth measurements (OD_{600}) for normal colony variant (NCV) and HQNO-induced small colony variant (SCV) *S. aureus* JE2 in response to treatment with CP-20 at varying concentrations. Dashed lines represent the average growth of DMSO-treated NCV and SCV *S. aureus*. Data are shown as mean ± SEM for three biological replicates.

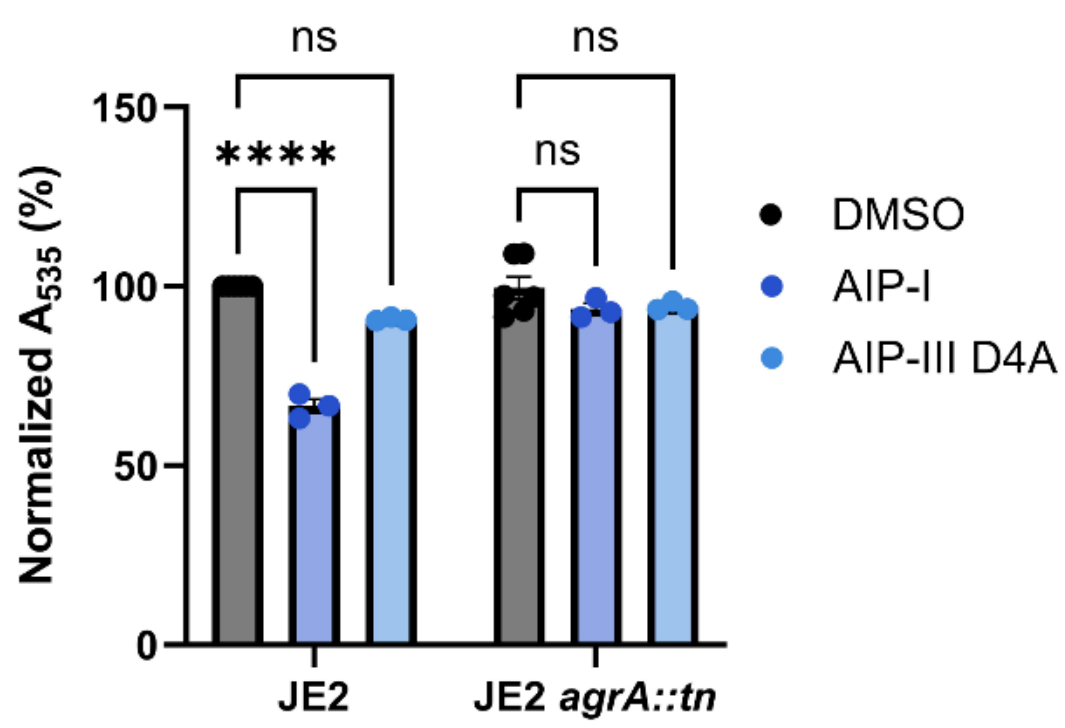

Figure S6. AIP-I inhibits *S. aureus* biofilm formation in an *agr*-dependent manner. Safranin staining-based biofilm measurements of biofilms formed by *S. aureus* JE2 and JE2 *agrA::tn*. Absorbance at 535 nm was used as a proxy for total adherent biomass. Absorbance values normalized to the absorbance at 535 nm for biofilms formed by DMSO-treated JE2. Strains were treated with DMSO (vehicle control), AIP-I (10 μM), or AIP-III D4A (100 nM). Data are shown as mean ± SEM for at least three biological replicates. **** $\alpha < 0.0001$ by a two-way ANOVA with Šídák's multiple comparisons test.

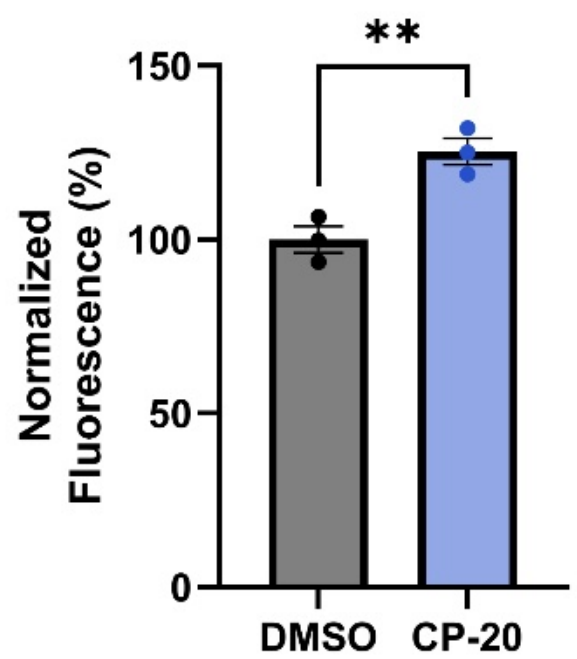

Figure S7. CP-20 weakly activates the SrrAB two-component system in *S. aureus*. Fluorescent data for the *S. aureus* SrrAB two-component transcriptional reporter system upon treatment with DMSO (vehicle control) or CP-20 (25 μM). The SrrAB reporter strain contains a plasmid encoding green fluorescent protein (GFP) under the control of the SrrAB-regulated *qox* promoter. Data are shown as mean ± SEM for three biological replicates. ** $p < 0.01$ by a Student's t-test.

References.

(1) Fey, P. D.; Endres, J. L.; Yajjala, V. K.; Widhelm, T. J.; Boissy, R. J.; Bose, J. L.; Bayles, K. W. A genetic resource for rapid and comprehensive phenotype screening of nonessential *Staphylococcus aureus* genes. *mBio* **2013**, *4* (1), e00537-00512. DOI: 10.1128/mBio.00537-12.

(2) Bosch, M. E.; Bertrand, B. P.; Heim, C. E.; Alqarzaee, A. A.; Chaudhari, S. S.; Aldrich, A. L.; Fey, P. D.; Thomas, V. C.; Kielian, T. *Staphylococcus aureus* ATP Synthase Promotes Biofilm Persistence by Influencing Innate Immunity. *mBio* **2020**, *11* (5), e01581-01520. DOI: 10.1128/mBio.01581-20.

(3) Rapun-Araiz, B.; Haag, A. F.; De Cesare, V.; Gil, C.; Dorado-Morales, P.; Penades, J. R.; Lasa, I. Systematic Reconstruction of the Complete Two-Component Sensorial Network in *Staphylococcus aureus*. *mSystems* **2020**, *5* (4), e00511-00520. DOI: 10.1128/mSystems.00511-20.

(4) Tal-Gan, Y.; Stacy, D. M.; Foegen, M. K.; Koenig, D. W.; Blackwell, H. E. Highly potent inhibitors of quorum sensing in *Staphylococcus aureus* revealed through a systematic synthetic study of the group-III autoinducing peptide. *J. Am. Chem. Soc.* **2013**, *135* (21), 7869-7882. DOI: 10.1021/ja3112115.

(5) Polaske, T. J.; Vulpis, T. D.; Nelson, A. E.; Zhao, K.; Blackwell, H. E. A Small Molecule that Inhibits the Quorum Sensing Receptor AgrC in *Staphylococcus aureus*. *J. Am. Chem. Soc.* **2026**, *148* (9), 9199-9204. DOI: 10.1021/jacs.5c21051.

(6) Solntsev, S. K.; Shortreed, M. R.; Frey, B. L.; Smith, L. M. Enhanced Global Post-translational Modification Discovery with MetaMorpheus. *J. Proteome Res.* **2018**, *17* (5), 1844-1851. DOI: 10.1021/acs.jproteome.7b00873.

(7) Tyanova, S.; Temu, T.; Sinitcyn, P.; Carlson, A.; Hein, M. Y.; Geiger, T.; Mann, M.; Cox, J. The Perseus computational platform for comprehensive analysis of (prote)omics data. *Nat. Methods* **2016**, *13* (9), 731-740. DOI: 10.1038/nmeth.3901.

(8) Duggan, S.; Laabei, M.; Alnahari, A. A.; O'Brien, E. C.; Lacey, K. A.; Bacon, L.; Heesom, K.; Fu, C.-L.; Otto, M.; Skaar, E.; McLoughlin, R. M.; Massey, R. C. A Small Membrane Stabilizing Protein Critical to the Pathogenicity of *Staphylococcus aureus*. *Infect. Immun.* **2020**, *88* (9), e00162-00120. DOI: 10.1128/iai.00162-20.

(9) Barnes V, L.; Heithoff, D. M.; Mahan, S. P.; House, J. K.; Mahan, M. J. Antimicrobial susceptibility testing to evaluate minimum inhibitory concentration values of clinically relevant antibiotics. *STAR Protocols* **2023**, *4* (3), 102512. DOI: 10.1016/j.xpro.2023.102512.

(10) Hughes, C. S.; Moggridge, S.; Muller, T.; Sorensen, P. H.; Morin, G. B.; Krijgsveld, J. Single-pot, solid-phase-enhanced sample preparation for proteomics experiments. *Nat. Protoc.* **2019**, *14* (1), 68-85. DOI: 10.1038/s41596-018-0082-x.

(11) Veling, M. T.; Reidenbach, A. G.; Freiberger, E. C.; Kwiecien, N. W.; Hutchins, P. D.; Drahnak, M. J.; Jochem, A.; Ulbrich, A.; Rush, M. J. P.; Russell, J. D.; Coon, J. J.; Pagliarini, D. J. Multi-omic Mitoprotease Profiling Defines a Role for Oct1p in Coenzyme Q Production. *Mol. Cell* **2017**, *68* (5), 970-977. DOI: 10.1016/j.molcel.2017.11.023.

(12) Yarlagadda, V.; Medina, R.; Wright, G. D. Venturicidin A, A Membrane-active Natural Product Inhibitor of ATP synthase Potentiates Aminoglycoside Antibiotics. *Sci. Rep.* **2020**, *10*, 8134. DOI: 10.1038/s41598-020-64756-0.

(13) Mitchell, G.; Gattuso, M.; Grondin, G.; Marsault, E.; Bouarab, K.; Malouin, F. Tomatidine inhibits replication of *Staphylococcus aureus* small-colony variants in cystic fibrosis airway epithelial cells. *Antimicrob. Agents Chemother.* **2011**, *55* (5), 1937-1945. DOI: 10.1128/AAC.01468-10.

(14) Eisenbraun, E. L.; Vulpis, T. D.; Prosser, B. N.; Horswill, A. R.; Blackwell, H. E. Synthetic Peptides Capable of Potent Multigroup Staphylococcal Quorum Sensing Activation and Inhibition in Both Cultures and Biofilm Communities. *J. Am. Chem. Soc.* **2024**, *146* (23), 15941-15954. DOI: 10.1021/jacs.4c02694.

(15) Jordan, S. C.; Hall, P. R.; Daly, S. M. Nonconformity of biofilm formation in vivo and in vitro based on *Staphylococcus aureus* accessory gene regulator status. *Sci. Rep.* **2022**, *12*, 1251. DOI: 10.1038/s41598-022-05382-w.

(16) Jenul, C.; Horswill, A. R. Regulation of *Staphylococcus aureus* Virulence. *Microbiol. Spectrum* **2019**, *7* (2), GPP3-0031-2018. DOI: 10.1128/microbiolspec.GPP3-0031-2018.
